# Using metabarcoding and droplet digital PCR to investigate drivers of historical shifts in cyanobacteria from six contrasting lakes

**DOI:** 10.1038/s41598-022-14216-8

**Published:** 2022-07-27

**Authors:** Maïlys Picard, Xavier Pochon, Javier Atalah, John K. Pearman, Andrew Rees, Jamie D. Howarth, Christopher M. Moy, Marcus J. Vandergoes, Ian Hawes, Samiullah Khan, Susanna A. Wood

**Affiliations:** 1 Coastal and Freshwater Group, Cawthron Institute, 98 Halifax Street East, Nelson, 7010 New Zealand; 2 Department of Biological Sciences, School of Biological Sciences, University of Waikato, Hamilton, 3216 New Zealand; 3 Institute of Marine Science, University of Auckland, Private Bag 349, Warkworth, 0941 New Zealand; 4 School of Geography, Environment and Earth Sciences, Victoria University of Wellington, PO Box 600, Wellington, New Zealand; 5 Department of Zoology, University of Otago, 340 Great King Street, North Dunedin, Dunedin, 9016 New Zealand; 6 GNS Science, 1 Fairway Drive, Avalon, Lower Hutt, 5011 New Zealand

**Keywords:** Microbiology, Molecular biology, Ecology, Environmental sciences, Limnology

## Abstract

The frequency and intensity of cyanobacterial blooms is increasing worldwide. Multiple factors are implicated, most of which are anthropogenic. New Zealand provides a useful location to study the impacts of human settlement on lake ecosystems. The first humans (Polynesians) arrived about 750 years ago. Following their settlement, there were marked landscape modifications which intensified after European settlement about 150 years ago. The aims of this study were to reconstruct cyanobacterial communities in six lakes over the last 1000 years and explore key drivers of change. Cyanobacterial environmental DNA was extracted from sediment cores and analysed using metabarcoding and droplet digital PCR. Cyanobacteria, including potentially toxic or bloom forming species, were already present in these lakes prior to human arrival, however their overall abundance was low. Total cyanobacteria abundance and richness increased in all lakes after European settlement but was very pronounced in four lakes, where bloom-forming taxa became dominant. These shifts occurred concomitant with land-use change. The catchment of one deteriorated lake is only moderately modified, thus the introduction of non-native fish is posited as the key factor driving this change. The paleolimnological approach used in this study has enabled new insights into timing and potential causes of changes in cyanobacterial communities.

## Introduction

Freshwater ecosystems throughout the world are currently experiencing an increase in frequency and intensity of cyanobacterial blooms^1–3^. Various causes have been identified, such as increased human disturbances including enhanced erosion caused by deforestation and land-use intensification, and major shifts in food-webs due to the introduction of non-native species (such as fish and macrophytes)^4,5^. A further compounding factor is climate change, which is resulting in warmer water temperatures, longer periods of stratification, greater frequency of severe weather events such as storms or droughts, and reduced length of ice cover, all of which have also been implicated in the rise of cyanobacterial proliferations^2,6–8^. Many bloom-forming species produce toxins collectively known as cyanotoxins, which can affect the reproduction and behaviour of aquatic organisms and have led to poisoning, or in extreme cases, death of humans and animals^9,10^. Considerable effort has therefore been placed on developing lake management and restoration plans for water bodies experiencing cyanobacterial blooms^11–13^. Unfortunately, historical records are very limited and monitoring programmes often start once a lake is already in an advanced state of degradation. There is uncertainty as to whether the recent increase in cyanobacterial blooms is entirely due to eutrophication and environmental change, or to a greater awareness and more monitoring programs contributing to these new bloom reports^14^. Increasing knowledge on how lake ecosystems, their catchments and regional climates have changed historically, and identifying links with cyanobacterial blooms, may help in the development of informed and successful restoration actions. New Zealand is an island nation in the South Pacific that has been isolated from all other landmasses for over 80 million years. The first humans to set foot on the mainland were from Polynesia (Māori), and only arrived around 1250–1350 AD^15–17^ . Following their arrival, there were marked landscape modifications in some regions of the country. For example, burning of native forests^18–21^ reduced overall coverage by about 50%^20,21^. Land clearance intensified with the first major wave of European settlement beginning in the 1840s^16^, coupled with farming practices which used fertilisers leading to nutrient runoffs, urbanisation releasing contaminants in waterways, and the introduction of many non-native freshwater species affecting native food-webs^22–24^.

Paleolimnology provides a suite of methods that allow inferences to be made about a lake’s historical condition—its past biological communities, water quality, and vegetation changes in and around the lake. For example, shifts in biological communities, such as midges, diatoms, or vegetation, can be indicative of past disturbance events^25–28^. Paleolimnological reconstructions have traditionally relied on microfossil indicators, however molecular methods targeting environmental DNA (eDNA) now allow a wider range of micro- or macro-organisms to be investigated in sediment cores, particularly soft-bodied organisms. While DNA can be extracted from the organism, it can also be recovered when it is bound to extracellular matrices in the sediment (exDNA)^29^. Recent work has shown that it is possible to use DNA from sediment (sedDNA), and ancient DNA from sediment (sedaDNA) to reconstruct the historical dynamics of various lake organisms including mammals, fish, and plants^30–33^. High-Throughput Sequencing (HTS) coupled with metabarcoding analyses allows for a large number of DNA sequences to be amplified directly from environmental samples and these can be used to characterise entire communities^34–37^. A recent study used metabarcoding to reconstruct cyanobacterial diversity over the last 200 years in Lake Greifensee and Lake Zürich, Switzerland^38^. Their results were congruent with an independent dataset obtained by microscopic identification of water samples for the last 40 years. Furthermore, quantitative PCR (qPCR) or droplet digital PCR (ddPCR) have also been successfully applied to paleolimnological studies for various targets including cyanobacteria and genes involved in cyanotoxin production in several countries^39–42^.

New Zealand has 3820 natural lakes greater than a hectare^43^, and due to its recent human settlement it provides a particularly useful location to study the ecological impacts of human settlement on lake ecosystem. It is possible to obtain sediment from pre-human times and reconstruct the past c. 1000 years with a relatively short sediment core (1–2 m) for most small lakes. Understanding the past of lakes is important because less than 200 of New Zealand’s lakes have regular water quality monitoring programmes in place, most of which have been underway for less than two decades. It is estimated that 46% of all lakes are eutrophic^44^ with many experiencing cyanobacterial blooms. The direct impact of anthropogenic activities is thought to be the main driver, but climate change may also be contributing. Over the last century, land temperatures have increased by 1 °C on average, droughts and floods have become more frequent and severe^44^.

The aims of this study were to reconstruct cyanobacterial communities in six New Zealand lakes over a period spanning approximately 1000 years using sediment cores, ddPCR and metabarcoding, and to identify key drivers that may have led to increases in cyanobacteria abundance or shifts in community structure. We hypothesised that: (1) cyanobacterial abundance and composition would start to shift during the period following Māori settlement in lakes where the vegetation cover in the catchment had changed. However, the most pronounced change would occur post European settlement with bloom-forming species becoming more prevalent in lakes which had experienced land use change; and (2) in lakes with little modification in vegetation cover in their catchment, the increases in abundance and changes in composition would be related to the presence of non-native carnivorous fish having a top-down effect on the food chain. While we acknowledge that climate change is likely to have some impact on the study lakes, we do not directly explore this in this study.

## Methods

### Sampling

**Figure 1:**
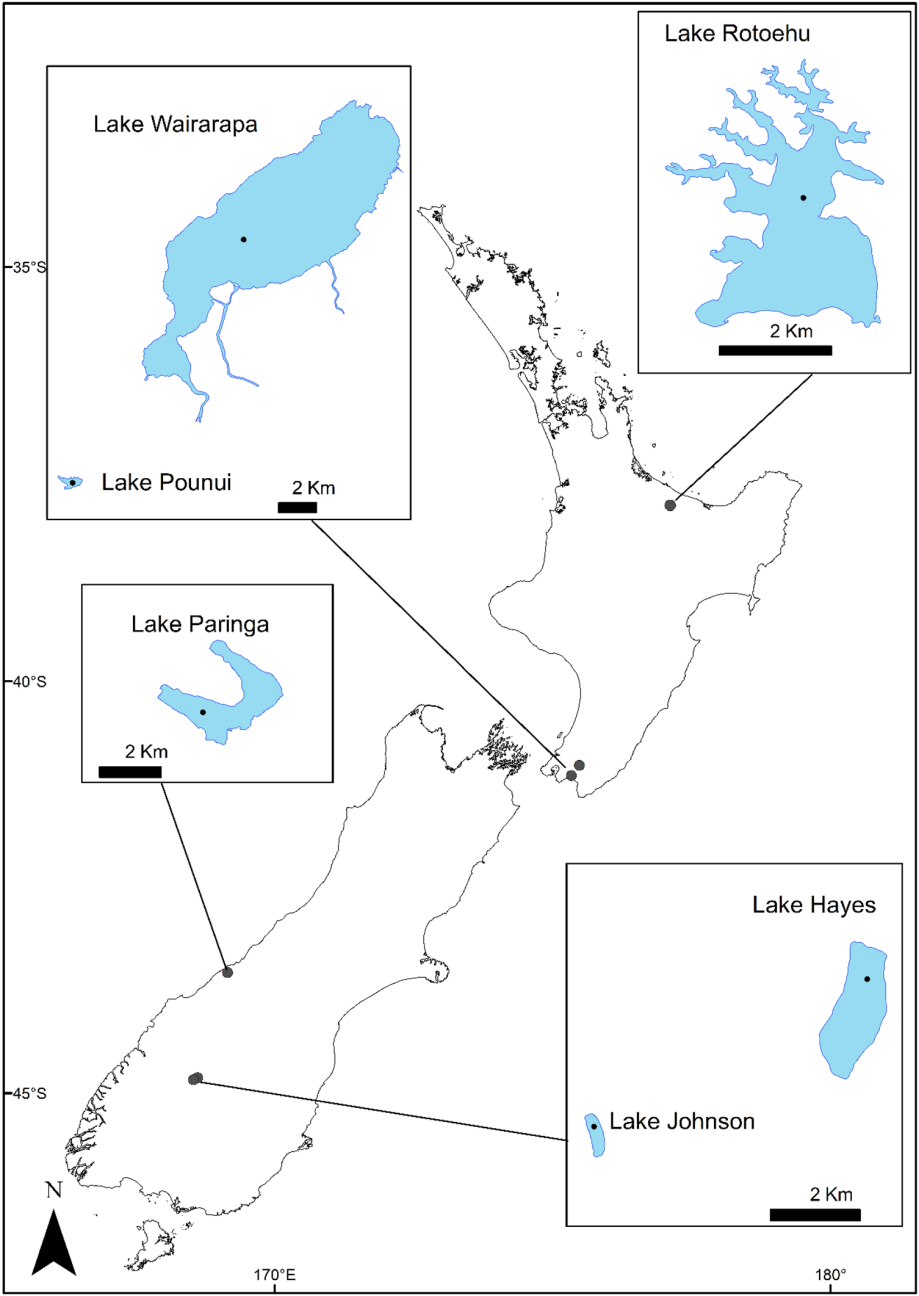
Location of the six study lakes, situated within the North and South islands of New Zealand and spanning 7° of latitudinal gradient. Black dots in each lake indicate coring sites. Map created using ArcGIS version 2.8 (https://www.esri.com) by Kati Doehring, Cawthron Institute.

Sediment cores were collected from six lakes in New Zealand: Rotoehu, Pounui, Wairarapa, Paringa, Hayes, and Johnson (Fig. 1, Table 1). These lakes were chosen because they have a wide variety of catchment modifications, span a range of trophic levels, have varying histories of cyanobacterial blooms, and contain different species of non-native fish (Table 1). The lakes span a range of climatic regions (Table 1).

**Table 1:** Lake characteristics.

	Paringa	Pounui	Rotoehu	Hayes	Johnson	Wairarapa
FENZ ID	46,725	229	40,188	54,190	53,707	1708
Latitude (S)	43°43′07.0"	41°20′40.0"	38°00′54.4"	44°58′28.6"	45°00′06.5"	41°13′31.6"
Longitude (E)	169°23′17.5"	175°06′51.8"	176°31′53.4"	168°48′40.7"	168°43′55.8"	175°12′58.0"
Rainfall (mm/month)	200–300	75–137	92–136	50–75	50–75	75–137
Temperature (°C/month)	7.4–15.6	8.9–17.7	7.8–17.9	3–15.8	3–15.8	8.9–17.7
Coring date	December 2017	August 2016	April 2019	November 2017	November 2017	June 2017
Core total length (m)	5.52	1.30	1.52	0.82	0.80	2.00
Lake elevation (masl)	16^45^	14^45,46^	296.9^46^	324.8^46^	392.1^46^	1.3^46^
Lake max depth (m)	52*	9.6^47^	13.5^46^	33^48^	27^46,49^	2.5^45,46^
Lake surface area (km^2^)	4.75^45^	0.46^45,46^	7.90^45,46^	2.74^45,49^	0.25^45,46^	77.37^45,46^
Lake total volume (km^3^)	0.29^46^	0.002^46^	0.035^46^	0.31^45^	0.002^46^	0.64^46^
Lake type	Glacial^46^	Tectonic^46^	Volcanic^46^	Glacial^46^	Glacial^46^	Riverine^46^
Mixing type	No data	Polymictic^47^	Polymictic^49^	Monomictic^48^	Monomictic^48^	Isothermal^49^
Current catchment area (km^2^)	79.09^45^	6.27^45^	73.85^45^	44^45^	1.90^45^	654.25^45^
Current catchment composition	Native forest and shrubs^46^	Native forest and a bit of pasture^46^	Pasture, urban, native forest^46^	Pasture and grasslands, urban, bit of native forest^46^	Mainly pastoral land, bit of native forest and shrubs^46^	Pasture, urban, native forest^46^
Trophic status—TLI	Oligotrophic (2020)*	Eutrophic (2014)^50^	Eutrophic (2018)^49^	Eutrophic (2018)^49^	Eutrophic (2018) ^49^	Supertrophic (2018)^49^
Reported cyanobacterial blooms	Not monitored	Yes^49^	Yes^49^	Yes^48,49^	Yes ^48^	Yes^50^
Non-native carnivorous fish species	Brown trout (*Salmo trutta*) in 1950s, Chinook/Quinnat salmon (*Oncorhynchus tshawytscha*) from 2011	Rainbow trout (*Oncorhynchus mykiss*) from 1938 to 1958 (Adam Canning pers. comm.), European perch (*Perca fluviatilis*) in the 1960s (Rawhiri Smith pers. comm.)	Rainbow trout (*O. mykiss*) around 1900^51^	Brown trout (*S. trutta*) and European perch (*P. fluviatilis*) from 1870s^52,53^	European perch (*P. fluviatilis*) from 1880s (Otago Fish & Game, unpublished data), rainbow trout (*O. mykiss*) from 1962^48^	European perch (*P. fluviatilis*) unknown date^45^

Note that the FENZ ID is related to the Freshwater Ecosystems of NZ (FENZ) geo-database, and the Trophic Level Index (TLI) is based on total nitrogen, total phosphorous, water clarity, and chlorophyll-a. Rainfall and temperature values are the highest and lowest mean monthly values over one year, data from NIWA^54^. Lakes are ordered from left to right by increasing lake trophic status.

*Lakes380 unpublished data.

A sediment core was retrieved close to the deepest point of each lake (Table 1) using an Uwitech gravity corer with 2 m-long, 90-mm diameter polyvinyl chloride barrels. The exception was Lake Paringa, where the sediment core was collected using a Mackereth corer^55^ and sealed within plastic barrels (50 mm internal diameter). All barrels were cleaned with 2% sodium hypochlorite (bleach) prior to coring. Core lengths ranged from 0.80 to 5.52 m (Table 1). After retrieval, the cores were sealed, stored at 4 °C and in darkness for up to 4 weeks until sub-sampling.

### Sample processing

In a room separate from where all DNA work was undertaken, the cores were split in half using a manual saw and a guillotine. They were photographed and described in detail to relate sediment type and colour and the presence of organic material. To prevent cross-contamination caused by the splitting of the cores, the top 2–3 mm of one half-core per lake was carefully removed with a sterile spatula. Sub-samples (c. 0.5 g) were taken from the centre of the half-core using a sterile spatula at various depths down the core. In general, sub-samples were taken every 1–2 cm in recent sediments and every 4–5 cm in older sediments (Supplementary Table S1), resulting in 206 sediment subsamples. Sub-samples were kept frozen (− 20 °C) and in the dark until DNA extraction. Sub-samples were also collected from a range of depths for pollen and charcoal analysis in all lakes as well as carbon-14 dating for Lakes Paringa and Pounui. These analyses are described elsewhere^47,56–58^ and a brief summary of methods is provided in Supplementary Table S2.

### DNA extraction and amplification

Each step of molecular analyses (DNA extraction, PCR or ddPCR set-up, template addition, PCR/ddPCR analysis) was conducted in separate sterile laboratories dedicated to these steps, with sequential workflow to ensure no cross-contamination. Rooms dedicated to DNA extraction, PCR set-up, or template addition were equipped with ultra-violet sterilisation which was switched on for at least 15 min before and after each use. The PCR/ddPCR set-up and template addition was always undertaken in laminar flow cabinets with HEPA filtration. Aerosol barrier tips (Axygen, USA for PCR or epT.I.P.S., Eppendorf, Hamburg, Germany for ddPCR) were used throughout.

Approximately 0.25 g of sediment from each sub-sample were weighed in the first tube provided in the DNeasy Power-Soil™ DNA Isolation Kit (QIAGEN, Germany) and exact weights recorded. Environmental DNA was extracted following the manufacturer’s protocol. DNA extraction was performed in batches of eight to ten samples, including a negative control every two batches which contained all the reagents but no sediment. DNA concentrations and quality were measured using a spectrophotometer (Eppendorf AG, Hamburg, Germany).

### Quantifications of total cyanobacteria

Droplet digital Polymerase Chain Reaction (ddPCR) was used to quantify total cyanobacteria from each sample using the CYAN 108F and CYAN 377R primers targeting an approximate 270 base-pairs (bp) region of the 16S rRNA gene^59^. All samples were diluted for quantification by ddPCR; dilutions ranged from 1/10 to 1/1000. The ddPCR was undertaken using a BioRad QX200 system. Each ddPCR reaction included 10 µL of 2 × BioRad QX200 ddPCR EvaGreen Supermix, 0.2 µL of each primer at 10 µM, 4 µL of diluted template DNA, and 7.6 µL of DNA/RNA-free water (Life Technologies) for a total reaction volume of 22 µL per well, loaded on a semi-skirted ddPCR 96-well plate. The BioRad QX200 droplet generator partitioned each reaction mixture into tens of thousands of nanodroplets by combining 20 µL of the mixture with 70 µL of Evagreen droplet oil. After processing, this resulted in a total nanodroplet volume of 40 µL, which was transferred to another semi-skirted ddPCR 96-well plate for amplification using specific thermo-cycling conditions (Supplementary Table S3). The plate was analysed on the QX200 droplet reader instrument. For each ddPCR plate run, at least one negative control (containing all reagents and DNA/RNA-free water instead of template DNA) and one positive control (genomic DNA extracted a cyanobacteria culture) were included. Droplet digital PCR droplet counts were fitted to a Poisson distribution by the QuantaSoft Analysis software (BioRad), resulting in target DNA concentration (gene copies/µL) which were then standardised to DNA gene copy numbers per gram of sediment using the following formula:

$${\text{Target}}\;{\text{concentration}}({\text{gene}}\;{\text{copies}}/{\text{g}}) = {\text{ }}\frac{{\frac{{nbcopies}}{{\mu L}}*\frac{{Total\;Mastermix\;volume\;\left( {22\mu L} \right)}}{{Template\;DNA\;volume\left( {4\mu L} \right)}}*dilution\;factor\left( {10 - 1000} \right)*DNA\;extraction\;volume\;\left( {100\mu L} \right)}}{{sediment\;sample\;weight\left( g \right)*\left( {1 - water\;content} \right)}}$$

### Reconstructing cyanobacterial communities with metabarcoding

Polymerase Chain Reactions (PCRs) were performed using the cyanobacteria-specific primers CYB359-F and CYB784-R^60^. These primers amplify an approximately 400 bp fragment of the V3-V4 regions of the cyanobacterial 16S rRNA gene. Sedimentary DNA samples were amplified in PCRs of 48 µL volume each, containing AmpliTaq Gold® 360 Master Mix (Life Technologies), 360 GC enhancer (Life Technologies), Bovine Serum Albumin (Sigma), primers at 10 µM, DNA/RNA free water (Life Technologies), and template DNA (Supplementary Table S3). Batches contained 20 samples, including a negative and positive control, PCR cycling conditions are detailed in Supplementary Table S3.

Amplicon products, including all negative extraction and PCR controls, were visualised on a 1.5% agarose gel electrophoresis stained with Red Safe DNA Loading Dye (iNtRON Biotechnology Inc, Kyungki-Do, Korea), and UV illumination to ensure amplification of a single ~ 400 bp product. PCR products were purified (Agencourt AMPure XP Kit; Beckman Coulter, CA, USA), quantified (Qubit® 2.0 Fluorometer; Invitrogen, CA, USA), diluted to 5 ng µL^−1^ and submitted for sequencing to Auckland Genomics (University of Auckland). Sequencing adapters and sample-specific indices were added to each amplicon via a second short round of PCR using the Nextera™ Index kit (Illumina, CA, USA). Amplicons were pooled into a single library and paired-end sequences (2 × 250 bp) were generated on a MiSeq™ instrument using the TruSeq™ SBS kit (Illumina, CA, USA). Sequence data were automatically demultiplexed using a MiSeq Reporter (v2), and forward and reverse reads were assigned to samples.

### Bioinformatic analyses

The R software^61^ and RStudio software^62^ were used for all bioinformatic and statistical analyses. The Tidyverse v1.3.0 and its associated packages^63^ were used for data manipulation and exploration. All plots were drawn using the ggplot2 package^64^ unless stated otherwise. Primer sequences were removed with Cutadapt^65^ (Anaconda environment adapted in R) allowing 1 bp of mismatch. The DADA2 package^66^ was used for general sequence quality assessment, quality profiles plots, and for the full amplicon workflow (quality filtering, merging of paired-end reads, dereplication, chimera identification, sample inference, and taxonomy assignment). Reads were truncated at 225 and 215 bp for forward and reverse reads respectively. The maximum number of “expected errors” (maxEE) per read was set at two for forward reads and four for reverse reads. Other parameters were set to default. The first 108 bp of the sequences were then used to calculate a parametric error matrix for forward and reverse reads each, which were checked for convergence. Sequences were dereplicated and sequence variants were inferred by pseudo-pooling based on their respective error matrix. Singletons were discarded and remaining paired-end reads were merged with a maximum mismatch of 1 bp and a required minimum overlap of 10 bp, producing a sequence table. Samples from all sequencing runs were then merged into one sequence table. Amplicon lengths were filtered so that only those within the range of 379 and 403 bp were retained and checked for chimeras using the consensus method, resulting in Amplicon Sequence Variants (ASVs). Taxonomy was assigned from Kingdom to Genus for each ASV using a training set from the SILVA database r138^67–69^. If the prediction of the SILVA classifier was estimated to be correct at 85% or more (min bootstrap), then the taxonomy for that rank was assigned to the ASV.

The phyloseq package^70^ was used to prepare the sequence table and associated information and undertake some analyses (merging, rarefaction with rarefy_even_depth, richness measures with estimate_richness). All non-bacterial reads (eukaryotes, mitochondria, chloroplasts) were removed from the dataset. Rarefaction curves were plotted using the vegan package^71^ to visualize sampling depth for each sample. Rarefaction of the entire dataset was undertaken at 10,400 reads per sample using phyloseq on all remaining bacterial reads through random sub-sampling with no replacement. Cyanobacterial-only ASVs were selected after rarefaction for further univariate analysis (richness). After rarefaction, some samples appeared to have no cyanobacteria ASVs because their very low numbers of cyanobacterial reads were removed during rarefaction. Multivariate analyses were therefore undertaken on unrarefied samples, where photosynthetic cyanobacterial reads counts were transformed to relative reads counts (%) per sample.

### Defining phases and age models

Pollen and charcoal data were used to assign specific occupation periods which are referred to throughout this study as: Pre-Human (PH), Evidence of Māori Settlement (EMS, from approximately 1300 AD), and Post-European Settlement (PES, from approximately 1840 AD) phases (Supplementary Figs. S1 to S5). The main markers for the start of the EMS were peaks in charcoal and bracken fern spores, both of which are indicators of landscape disturbance^15,20^. The primary indicator for the start of the PES phase was the appearance of exotic plant pollens such as the Monterey pine (*Pinus radiata*), sheep’s sorrel (*Rumex acetosella*), and willow (*Salix* spp.)^72^. The catchment of Lake Paringa is relatively undisturbed and for this lake the palynology did not provide any useful information on the start or finish of the eras therefore the age model was used to assign these (see below).

Age models from ^14^C were developed in other studies for the sediment cores of Lake Pounui and Lake Paringa^47,56^ (presented in Supplementary Tables S4 and S5) and correlated and applied to the cores collected in this study. The cores from Lakes Rotoehu, Hayes, Johnson, and Wairarapa were not dated. In Lake Rotoehu, an approximately 5-cm tephra layer corresponded to the 1886 eruption of Mount Tarawera provides a defined point in time.

### Statistical analyses

Cyanobacteria abundances (number of 16S rRNA gene copies per gram of sediment) from ddPCR data were reconstructed across lakes. The number of photosynthetic cyanobacteria (also now commonly called oxyphotobacteria) ASVs (species richness) from metabarcoding data were calculated and plotted as downcore profiles; the number of species within each Order and Class (photosynthetic or not) were plotted as barplots, and the relationship between cyanobacteria abundance and photosynthetic richness for individual lakes was assessed using scatterplots and Spearman’s correlation.

Three binary categories of perturbations were assigned to each sample. The categories were: (1) land clearance as the start of large-scale vegetation disturbance—indicated by peaks of charcoal and/or bracken fern spores, which were indicative of burning, (2) land-use change – characterised by increases in pollen from non-native grasses and/or pine during the PES phase, and (3) presence of carnivorous non-native fish (trout or perch). Catchment-related perturbations were observed in all lakes apart from Lake Paringa, indicated by increased in charcoal, bracken spores, and/or terrestrial non-native pollen (Supplementary Figs. S1 to S5). When the sediment cores were dated, the dates of non-native fish introduction in each lake were determined directly or indirectly from historical documentation or oral historical reports (Lake Paringa^56^; Rawhiri Smith pers. comm. for Lake Pounui). Where no dating was available (Rotoehu, Hayes, Johnson, Wairarapa), non-native fish introduction was assumed to be associated with the start of the PES phase.

Generalised least squares (GLS) models were then used to investigate the drivers associated with changes in historical cyanobacteria total abundance and photosynthetic richness. GLS were chosen over other statistical methods to account for the temporal autocorrelation between proximate down-core samples^73^. Five candidate predictor variables were initially considered (Supplementary Table S6): Phase (three levels: PH, EMS, and PES), Non-native Fish (binary), Native Vegetation Change (binary), Land-Use Intensification (binary) and Lake (six levels: Hayes, Johnson, Paringa, Pounui, Rotoehu, and Wairarapa). Because all predictors were highly correlated and collinear, in particular non-native fish presence and land-use intensification, their effects were summarised by the three phases: PH, EMS—encompassing the beginning of native vegetation clearance, and PES—encompassing non-native fish introduction and land-use intensification. Though not all lakes were affected by these pressures (Lakes Paringa and Pounui have not undergone significant changes in their catchment), this proved to be the best option for modelling purposes. Models were fitted using Phase and Lake as fixed effects and with a continuous first-order autoregressive process (corCAR1), with sub-sample core depth nested within Lake to correct for autocorrelation. Because cyanobacteria abundances consisted of strictly positive and right-skewed continuous data while richness was mildly right-skewed, they were log and square-root-transformed, respectively. Model assumptions of normality, homogeneity of variances and independence were checked by inspecting normalised residuals, including using autocorrelation function plots. Only photosynthetic cyanobacteria were selected for the multivariate analysis.

Shared ASVs across the dataset, across lakes, across phases, and across phases within lakes were investigated, and visualised using Venn diagrams^74^. Changes in relative read abundance in the cyanobacterial community structure were plotted for each phase within each lake at genus level using stacked barplots. To further understand specific changes in cyanobacteria versus picocyanobacteria across time and lakes, all ASVs identified at Genus level were assigned to a cell diameter factor: either > 3 µm, or < 3 µm (picocyanobacteria), as a well as a toxin category (none or potentially toxic, Supplementary Table S7). Community changes at genus level were visualised with barplots and aligned with dendrograms using the packages rioja^75^ and ggdendro^76^. Variations in ASV community structure were then visualised using principal coordinates analyses ordination plots (PCoA) based on Bray–Curtis dissimilarities using the vegan package^71^. Difference in community structure were explored using a distance-based permutational analysis PERMANOVA^77^ based on Bray–Curtis dissimilarities of the relative abundance community data and 999 permutations. The experimental design consisted of Lake and Phase as fixed factors^78^. Significant terms were then investigated using pair-wise comparisons with the PERMANOVA t-statistic and 999 permutations. Tests for homogeneity of multivariate dispersions based on Bray–Curtis distances^78^ were used to compare distances to the multivariate centroids between Lake, and between Phase within individual lakes.

## Results

### General richness patterns

A total of 21,545 bacterial ASVs were recovered from the 208 samples sequenced. Of these, 1361 corresponded to all cyanobacteria and 1199 to photosynthetic cyanobacteria. After rarefaction (10,400 reads), a total of 1144 cyanobacterial ASVs remained, of which 928 were photosynthetic cyanobacteria. Several samples were discarded because they either did not have enough sequence reads to meet the rarefaction threshold (Supplementary Fig. S6, two samples), or they did not have any cyanobacterial reads following rarefaction (thirteen samples), or any photosynthetic cyanobacterial reads (further five samples).

Cyanobacterial ASVs were assigned to two different classes: Vampirovibrionia (previously Melainabacteria candidatus^79^, non-photosynthetic cyanobacteria) and Cyanobacteria (photosynthetic cyanobacteria). Cyanobacteria ASVs were found in every lake and were amplified in 193 instances in the rarefied dataset (i.e., 93.7% of investigated [n = 206] samples). There were no common photosynthetic cyanobacteria ASVs across all samples, with only three ASVs belonging to the genus *Cyanobium* found in all lakes. When aggregating all samples by phase, there were 34 shared ASVs across the three phases (PH, EMS, PES), belonging to the genera *Cyanobium* (25 ASVs), *Dolichospermum* (2 ASVs), *Microcystis* (4 ASV), *Synechocystis* (1 ASV), and 3 ASVs unassigned at Genus level (1 unknown Nostocales, 1 unknown Cyanobacteriales and 1 unknown Nostocaceae). An assessment of individual lakes showed that in Lakes Hayes and Johnson a single ASV was found in all samples (a different one in each lake), taxonomically assigned to the genus *Cyanobium*. In all lakes the highest number of shared ASVs was between the PES and EMS phases (Supplementary Fig. S7). Lake Wairarapa was the only lake that did not have a core community across all phases (Supplementary Fig. S7). Shared taxa differed from one lake to the other, though they belonged mainly to the picocyanobacteria genus *Cyanobium*. Total Cyanobacteria, Vampirovibrionia and (photosynthetic) Cyanobacteria ASVs varied among lakes, with the highest numbers recorded in Lake Johnson (Table 2). A total of 47.8% cyanobacterial ASVs were unclassified at Genus level (493 out of 1031), including 297 ASVs that corresponded to photosynthetic cyanobacteria.

**Table 2:** Cyanobacterial amplicon sequence variants (ASVs) per lake.

	Paringa	Pounui	Rotoehu	Hayes	Johnson	Wairarapa	Total
Number of samples	29	36	29	31	37	30	187
Total Cyanobacteria	116	134	254	272	359	142	1034
Cyanobacteria	80	115	225	223	280	129	839
Vampirovibrionia	36	19	29	49	79	13	195

Results are given for total cyanobacteria and per Class.

The majority of Vampirovibrionia ASVs could only be assigned at Order level (Caenarcaniphilales, Gastranaerophilales, Obscuribacterales, and Vampirovibrionales). Vampirovibrionia ASVs were found in every lake but only occurred in 104 out of the 193 samples and their richness differed greatly between lakes (> 40 ASVs in Lake Hayes and Johnson and < 40 ASVs in the other lakes, Supplementary Figs. S8 and S9). The Order Gastranaerophilales was the most common (7–72 ASVs per lake), followed by Obscuribacterales (4–11 ASVs per lake). The other two Orders (Caenarcaniphilales and Vampirovibrionales) were poorly represented and were not found in all lakes (Supplementary Fig. S9). Due to the lack of ecological knowledge on Vampirovibrionia, no further analysis was conducted.

The rarefied cyanobacterial photosynthetic dataset was composed of 8 orders, 21 families, and 51 genera across all lakes and all samples. The most common Cyanobacteria Order was Synechococcales, followed by Cyanobacteriales (formerly Nostocales; Supplementary Fig. S8). The number of Synechococcales ASVs was variable across lakes by an order of ten (55–153 ASVs per lake), and it was similar for Cyanobacteriales (14–120 ASVs per lake). All other photosynthetic cyanobacteria Orders were poorly represented (≤ 4 ASVs per lake) and were not detected in all lakes.

### Cyanobacterial richness and abundance profiles

**Figure 2:**
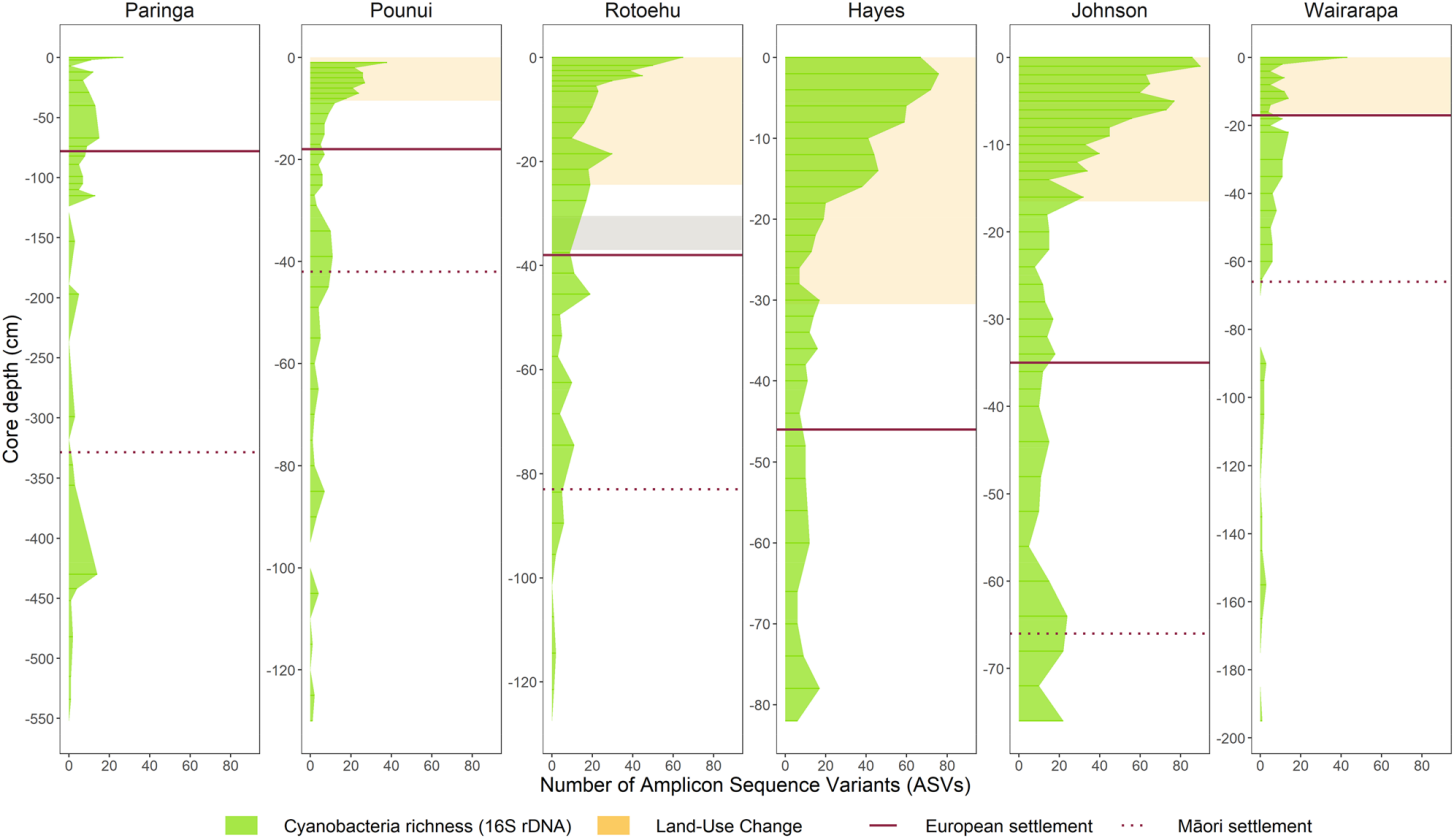
Richness of photosynthetic cyanobacterial Amplicon Sequence Variants (ASVs) from metabarcoding data, cyanobacterial 16S rRNA gene. The horizontal lines separate the three phases, pre-human (PH; bottom), evidence of Māori settlement (EMS; middle), and post European settlement (PES; top); note: the sediment core from Lake Hayes does not include any PH phase. Yellow shades indicate the depths associated with land-use intensification in relevant lakes and the grey shade indicates the 1886 Tarawera tephra layer in Lake Rotoehu.

The richness (number of ASVs) of photosynthetic cyanobacteria increased significantly in all lakes during recent times compared to their historical levels (Fig. 2). Overall, Lakes Hayes and Johnson had the highest richness and Lake Wairarapa the lowest (Figs. 2 and 4). Shifts in richness after European arrival were particularly notable in lakes known to currently experience cyanobacterial blooms (Hayes, Johnson, Pounui, Rotoehu). This was particularly obvious in Lake Rotoehu (PH = 0–6 ASVs, PES = 9–65 ASVs), while Lakes Hayes and Johnson displayed a very high richness in recent times (80–90 ASVs at the top of the core). Despite having moderate ‘European land-use’ in its catchment, Lake Pounui also showed an increase in richness. Timing of shifts were different for Lakes Pounui (~ 1900s) and Paringa (~ 1700s). No specific dates could be approximated for Lakes Rotoehu, Hayes, Johnson, and Wairarapa due to the absence of age models, though the increase in richness coincided with land-use intensification in Lake Johnson. The richness profile of Lake Wairarapa was more consistent with the timing of phases, with a slight increase to 8 ASVs on average after EMS until near the top of the core, with a marked increase in the top sample (43 ASVs).

**Figure 3:**
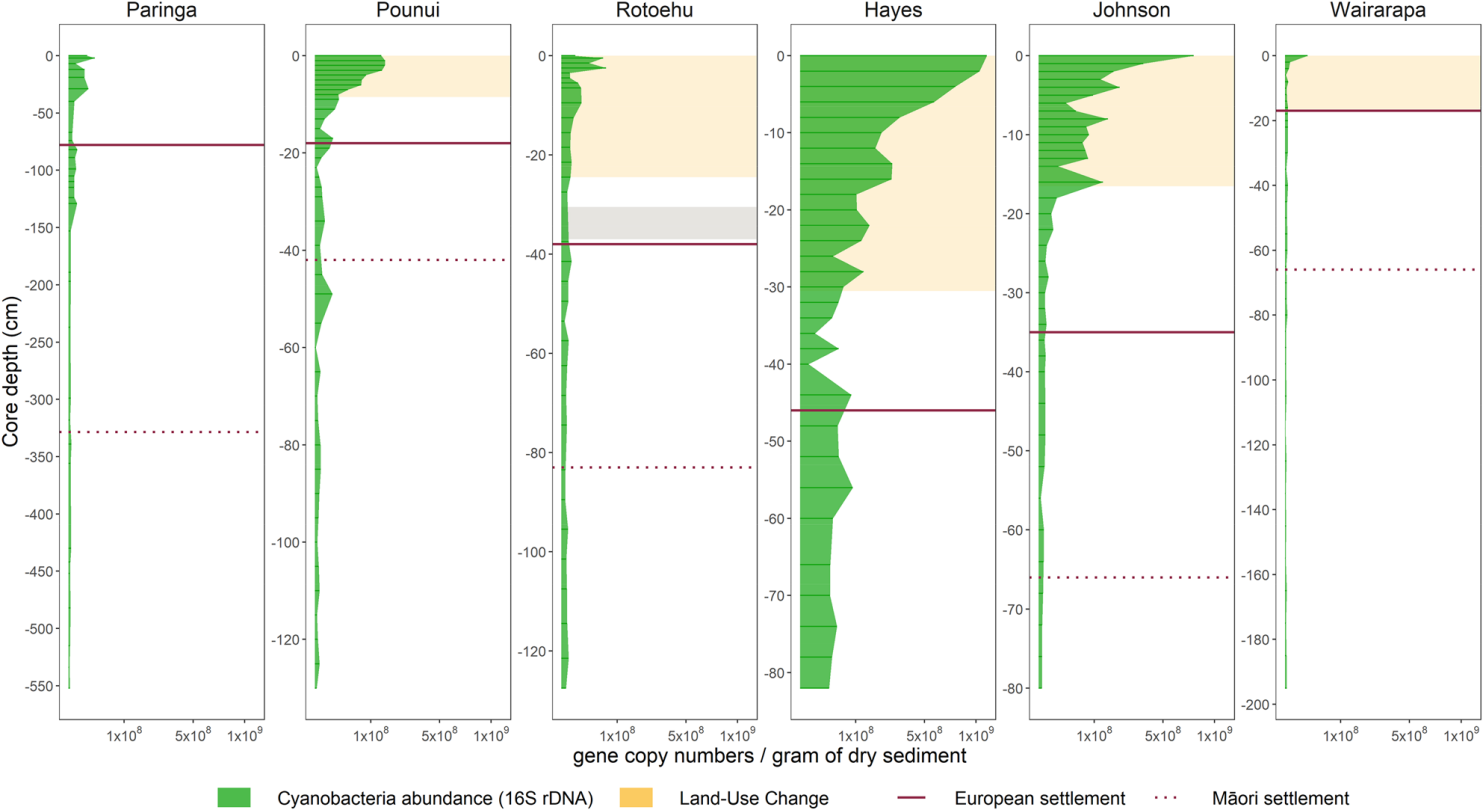
Cyanobacterial concentrations from droplet digital PCR data. Sediment depth profiles of cyanobacterial 16S rRNA gene copy numbers per gram of dry sediment, y-axis shown on square-root scale. The horizontal lines separate the three phases, pre-human (PH; bottom), evidence of Māori arrival (EMS; middle), and post European arrival (PES; top); note: the sediment core from Lake Hayes does not include any PH phase. Yellow shades indicate the depths associated with land-use intensification in relevant lakes, the grey shade indicates the 1886 Tarawera tephra layer in Lake Rotoehu.

**Figure 4:**
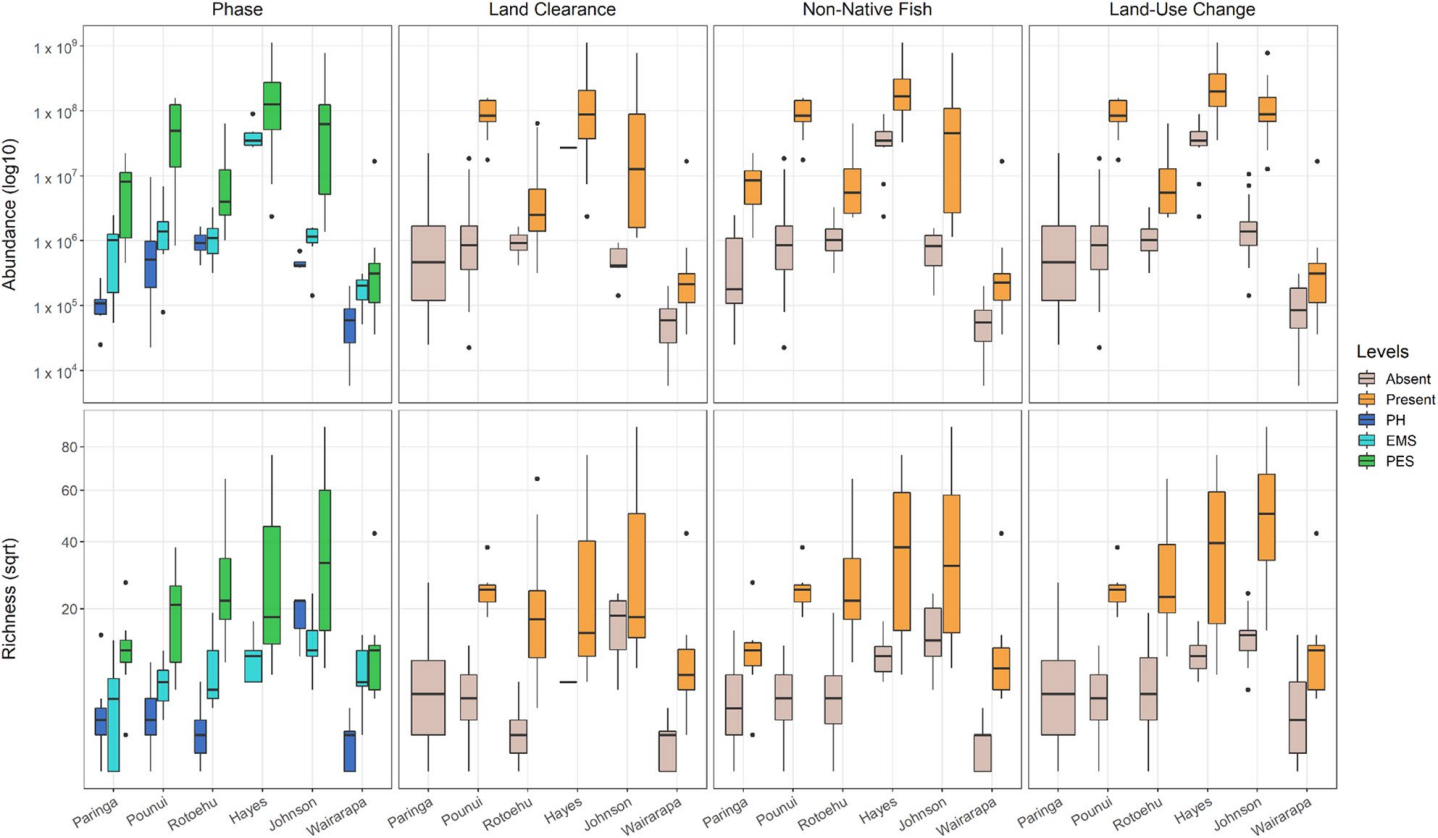
Differences in each response (cyanobacteria abundance or richness) depending on the lake and predictor variable. Cyanobacteria abundance was measured by droplet digital PCR (ddPCR–16S rRNA gene copy numbers/gram of dry sediment, log10 y-axis scale) while cyanobacteria richness was calculated from metabarcoding data (number of photosynthetic cyanobacteria Amplicon Sequence Variants per sample, square-root y-axis scale). PH = Pre-Human, EMS = Evidence of Māori Settlement, PES = Post European Settlement.

Cyanobacterial abundance (16S rRNA gene copy numbers per gram of dry sediment) also increased in all lakes during recent times compared to historical levels (Fig. 3). These increases were particularly prominent in Lakes Johnson and Pounui (up to ~ 6,000 times their historical levels), medium in Lakes Hayes, Rotoehu, and Paringa (around 500 times their historical levels), and a slight increase in Lake Wairarapa, which was mostly in the surface sample. Timing of shifts in cyanobacterial abundance were different for all lakes: Lake Paringa suddenly shifted in the mid 1900’s, Lake Pounui underwent a steady though steep increase post European arrival, while Lake Hayes and Johnson steadily increased with land-use intensification. Increase in abundance were more recent for Lakes Rotoehu and Wairarapa (top of the cores). Lastly, though the sediment core of Lake Hayes did not include the PH phase, its levels of cyanobacterial abundances during EMS were up to 1000 times higher than in the other lakes at the same time (Figs. 3 and 4).

When all lakes were combined, richness was positively correlated with 16S rRNA cyanobacterial gene copy numbers (r = 0.74, *p* < 0.001). Relationships were also significant when each lake was analysed individually (from R = 0.49 for Lake Wairarapa to R = 0.82 for Lake Pounui, all *p* < 0.01; Supplementary Fig. S10).

### Drivers of shifts in cyanobacterial community

Both cyanobacteria abundances and photosynthetic richness tended to increase with land clearance, land-use change, and the introduction of non-native (carnivorous) fish (Fig. 4). Generalised least squares (GLS) models only incorporated Phase and Lake since the other predictors were highly correlated. These models showed a significant effect of Phase on cyanobacteria abundance and richness (*p* < 0.01); compared to EMS, richness and abundance were significatively higher during PES and lower during PH (Table 3, Fig. 4). When comparing all lakes to Lake Paringa (the most pristine lake), GLS models revealed that all lakes had a significantly higher abundance than Lake Paringa apart from Lake Wairarapa which had a significantly lower abundance (Table 3, Fig. 4). Only Lakes Johnson and Hayes had a significantly higher richness (Table 3). When comparing lakes to one another with pairwise comparisons, the models revealed that overall, Lakes Hayes, Johnson and Pounui had a significantly higher 16S rRNA gene copy abundance than Lakes Paringa and Wairarapa (Supplementary Table S8), while Lake Johnson had a significantly higher richness than Lakes Paringa and Wairarapa (Supplementary Table S9).

**Table 3:** Results of the generalised least squares (GLS) models for cyanobacteria abundance (from droplet digital PCR data) and richness (from metabarcoding data).

Predictors	Estimates	log(Abundance) CI	p	Estimates	sqrt(Richness) CI	p
(Intercept)	12.91	12.32–13.49	**< 0.001**	1.98	1.29–2.67	**< 0.001**
Phase PES	1.64	1.04–2.24	**< 0.001**	1.04	0.39–1.69	**0.002**
Phase PH	− 1.13	− 1.75–− 0.51	**< 0.001**	− 1.05	− 1.77–− 0.32	**0.005**
Lake Pounui	4.28	3.32–5.25	**< 0.001**	0.62	− 0.49–1.73	0.27
Lake Rotoehu	1.62	0.68–2.57	**0.001**	0.90	− 0.21–2.01	0.11
Lake Johnson	1.44	0.60–2.28	**0.001**	2.57	1.27–3.87	**< 0.001**
Lake Hayes	1.13	0.29–1.97	**0.009**	1.76	0.47–3.05	**0.008**
Lake Wairarapa	− 1.08	− 1.86–− 0.30	**0.007**	0.21	− 0.79–1.20	0.68
Observations		209			206	

PH = pre-human, EMS = evidence of Māori settlement, PES post European settlement. PES and PH were compared to EMS since the Lake Hayes core does not have a PH phase, while all lakes were compared to Lake Paringa (most pristine lake).

Significant *p* values (*p* < 0.05) are shown in bold.

### Historical and spatial variability in cyanobacteria community structure

**Figure 5:**
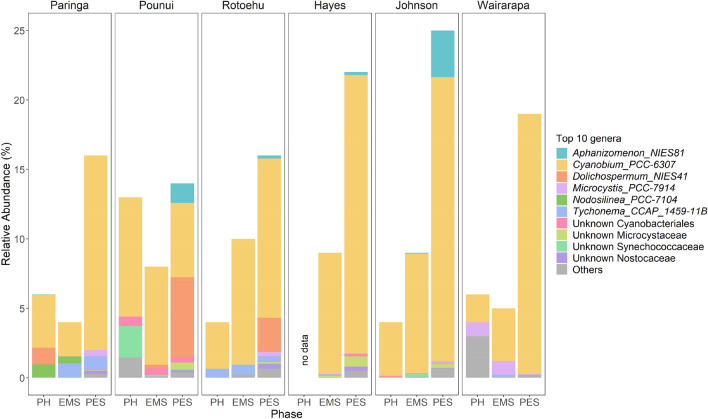
Relative abundance of the top 10 most abundant photosynthetic cyanobacterial genera across all lakes, less abundant genera are grouped as “Others”. All samples were grouped by Phase within each lake. PH = Pre-Human; EMS = Evidence of Māori Settlement; PES = Post-European Settlement. The sediment core from Lake Hayes does not include any PH phase.

**Figure 6:**
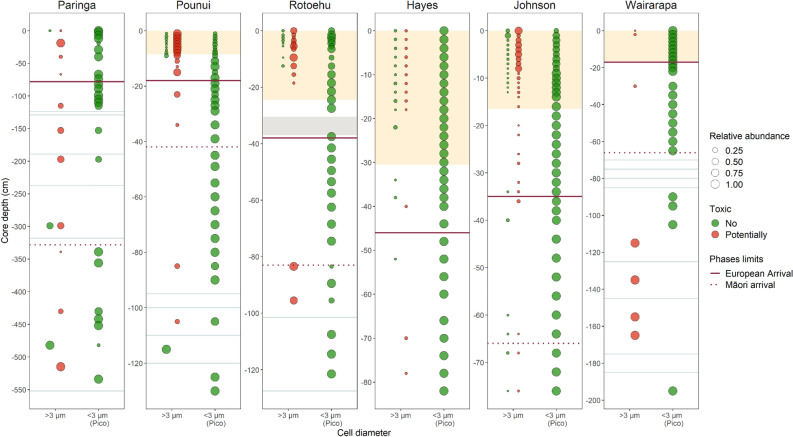
Relative abundance profiles of photosynthetic cyanobacterial genera classified by size (> 3 µm or < 3 µm—picocyanobacteria) and potential toxin production. Relative abundance is plotted as a continuous variable and the legend gives an indication of size for a set of abundances. Potential for toxin production of each genus was inferred from the literature. Phase delimitation is indicated by horizontal purple straight and purple dotted lines (see legend). Horizontal grey lines indicate samples with no cyanobacteria sequences after rarefaction. Yellow shades indicate land-use intensification in relevant lakes. Grey shade in the Lake Rotoehu core indicates the Tarawera tephra (1886).

Photosynthetic cyanobacterial reads were selected from the unrarefied dataset for the remainder of the analyses (n = 190 samples). *Cyanobium*, a picocyanobacterial genus, dominated the cyanobacterial community in almost all lakes and phases (Figs. 5 and 6). Other picocyanobacteria genera, such as *Synechocystis*, were present intermittently in low abundance in Lakes Pounui, Hayes, and Johnson (mainly PES). The main non-picoplanktonic genera were the potential toxigenic *Aphanizomenon*, *Microcystis*, and *Dolichospermum* and these became dominant in Lakes Johnson and Pounui in in the PES phase (Figs. 5 and 6). These were found in at least one sample in most lakes. *Aphanizomenon* and *Microcystis* were found in all lakes and mostly in the PES phase; *Dolichospermum* was restricted to Lakes Pounui (PES), Rotoehu (PES), Johnson (PES) and Paringa (PH), although relative abundances differed greatly among lakes. Other relatively common pelagic genera were only found in specific lakes: *Geminocystis* in Lake Hayes (PES), Johnson (PES and PH), and Rotoehu (PES), *Tychonema* in all lakes except Lake Pounui. Some benthic taxa were also identified, such as *Phormidium* and *Geitlerinema* in Lake Wairarapa, *Calothrix* in one sample from Lake Rotoehu (EMS), *Nodosilinea* in Lakes Paringa (EMS + PH, one sample each), Johnson (PES), and Wairarapa (PES, one sample), and *Oscillatoria* in Lake Paringa (EMS). Some potentially interesting taxa (high enough relative abundance) could unfortunately not be identified down to genus level, especially in Lakes Pounui and Hayes (Unknown Cyanobacteriales, Microcystaceae, and Synechoccaceae, Fig. 5). Overall, toxic taxa were present in all lakes prior to human arrival, however their relative abundance increased in Lakes Hayes, Johnson, Pounui, and Rotoehu in the PES phase. They became continuously occurrent from European arrival in Lakes Pounui and Johnson, and shortly after land-use intensification in Lakes Rotoehu and Hayes. In general, picocyanobacteria relative abundance remained constant, although slight decreases were observed in Lakes Johnson and Pounui as potentially toxic taxa became dominant (Fig. 6, Supplementary Fig. S11). The CONISS analysis showed that timings of shifts in the community composition varied depending on the lakes, though the main shift seemed to coincide with land-use intensification for Lakes Rotoehu, Hayes, and Johnson (Supplementary Fig. S11).

**Figure 7:**
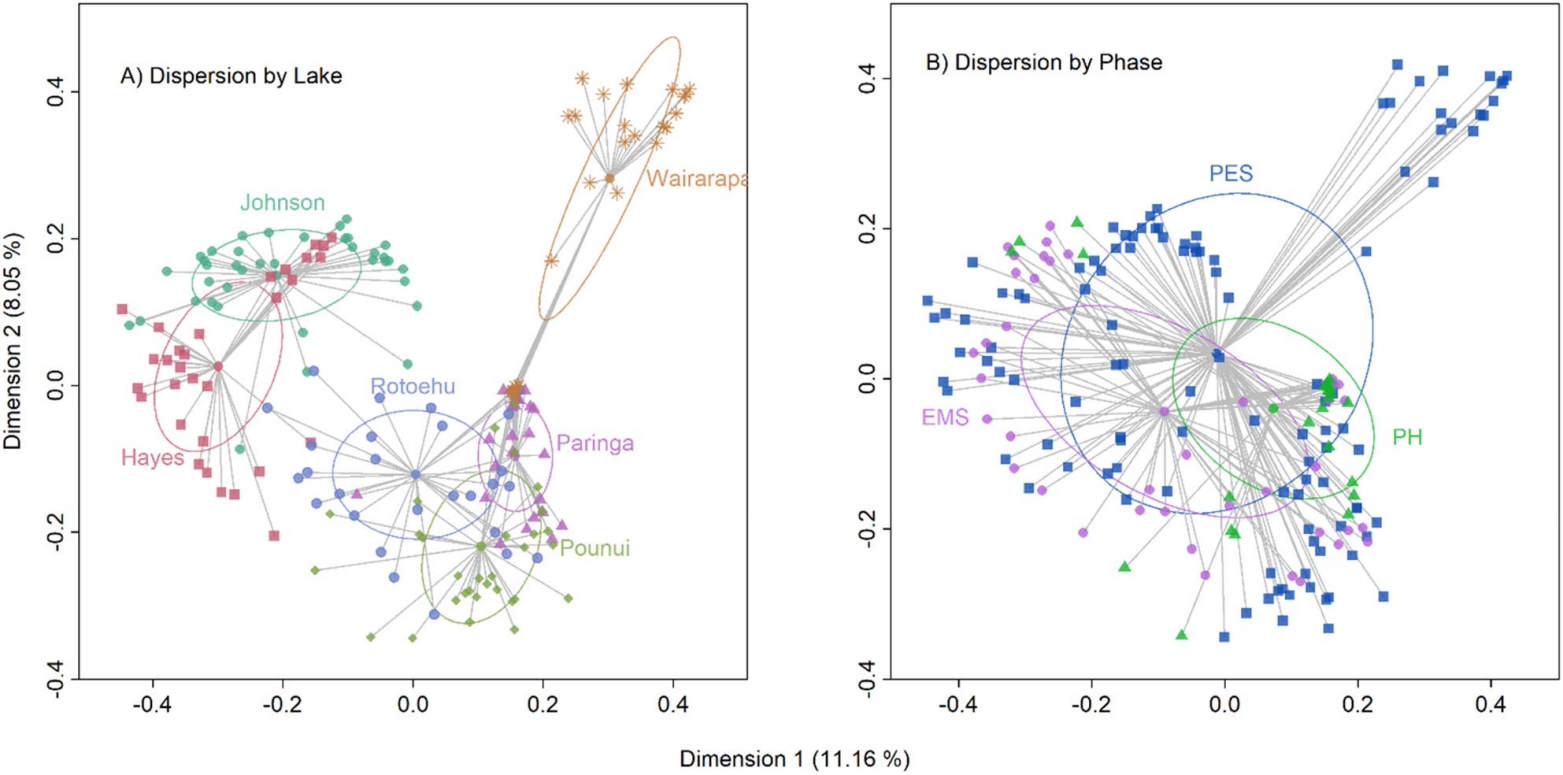
Principal coordinates analysis (PCoA) ordination plots of cyanobacterial communities based on Bray–Curtis dissimilarities for all six study lakes (**A**) and all phases (**B**). The ellipses indicate the mean distance from centroids, whereas the vectors show the distance to the centroid of each group.

The PERMANOVA test showed that there were significant differences in community structure across lakes (F_189,5_ = 11.726, *p* < 0.01), and pairwise comparisons revealed that all lakes were significantly different from one another (*p* < 0.01). The test for homogeneity of multivariate dispersions showed that these differences could be due to differences in the mean distance from lake centroids among lakes (F_184,5_ = 7.5613, *p* < 0.001). The PCoA ordination revealed three distinct clusters: Hayes and Johnson; Pounui, Paringa and Rotoehu; and Wairarapa by itself (Fig. 7A). Only Lake Hayes’s dispersion was significantly different from Lakes Rotoehu, Paringa, and Pounui (Tukey’s post-hoc test on homogeneity of multivariate dispersion, *p* < 0.05).

When data from all lakes was combined, there was no clear evidence of a differentiation of cyanobacterial assemblages by phase (Fig. 7B). When each lake was analysed individually, phases tended to be quite distinct and significatively different from one another (Supplementary Fig. S12, Supplementary Tables S10 and S11). All phases were different from one another in Lakes Pounui, Rotoehu, Hayes and Johnson; the PH phase was not different from EMS only in Lakes Paringa and Wairarapa (Supplementary Table S10). While some of the results may be affected by heterogeneous dispersion (Supplementary Table S11), the data indicate that the cyanobacterial community changed Post-European settlement (PES vs. EMS) in all lakes except Lake Pounui and Lake Rotoehu (Supplementary Fig. S12, Supplementary Tables S10 and S11).

## Discussion

### General patterns

We inferred a large diversity of cyanobacteria present within our study lakes. Interestingly, many ASVs were identified as picocyanobacteria (small—usually less than 3 µm, unicellular cyanobacteria), and in particular *Cyanobium*, regardless of lake or phase. It is unclear whether their high numbers are due to; (1) primer biases (i.e. the primers preferentially bind to these taxa and/or these taxa have higher 16S rRNA copies in the genome compared to other cyanobacteria), (2) more variability in their 16S rRNA sequences (which resulted in our bioinformatics pipeline separating them as different ASVs), (3) better cell or DNA preservation, or (4) if they are actually present in high concentrations in the environment. It is also possible that some cyanobacteria sequences are wrongly assigned as *Cyanobium* in the SILVA database, leading to an over-representation of this genus in our results. Given their small size and difficulties with identifying them, it is also highly likely they are commonly overlooked in traditional light microscopy analysis of lake samples^80^. A study of cyanobacterial diversity in 143 New Zealand lakes identified an equally high number of operational taxonomic units and associated sequence reads of picocyanobacteria and suggested that this group has been overlooked in most limnological studies in this country^81^. Other recent ecology studies^82,83^ have highlighted similarly diverse freshwater autotrophic picoplankton communities and suggested a need for further characterisation with particular regard to their response to environmental change. Although relative abundances of picocyanobacteria remained relatively constant across phases, there were differences in picocyanobacteria structure among lakes and between phases at the ASV level. Because so little is known about the ecology of these taxa, it is not feasible to make ecological inferences based on these shifts. Enhancing knowledge on their ecology would greatly assist in understanding the observed changes and may allow these taxa to be used as early indicators of change in lake ecosystems.

Potentially toxic genera and/or well-known bloom-forming species (e.g., *Microcystis*, *Dolichospermum*, *Aphanizomenon*, or *Phormidium*) were present in all lakes including before human arrival (except Lake Hayes, no pre-human period reconstructed). This corroborated previous findings that potentially toxic cyanobacteria have been present historically in lakes, albeit in low abundance or sporadically^42,84^. However, while bloom-forming species were occasionally common in some samples in pre-human times according to the metabarcoding data, this does not necessarily mean they were actually abundant in the lake. Rather, this could be an artifact due to few cyanobacteria sequences in the samples, since metabarcoding relies on relative abundance. This is reinforced by the ddPCR data which does not indicate high abundance of cyanobacteria in the pre-human period. Furthermore, shifts to dominance by potential bloom-forming species were only observed in recent times in lakes that are presently eutrophic (Pounui, Rotoehu, Hayes, Johnson), a trend which was reflected by an increase in total cyanobacterial abundance in the same lakes. Lake Wairarapa was the only exception, having general low levels of cyanobacteria despite its current supertrophic status.

Some non-photosynthetic cyanobacteria (Vampirovibrionia Class) were identified by the metabarcoding data. They mostly occurred in the top half of the cores, and could generally only be identified at Order level. It is likely that they were also detected by the ddPCR assay, and they may account for some of the observed increases in abundance and richness. Further research is needed to enhance knowledge on their ecological role.

### Lake-specific cyanobacteria histories

Total cyanobacterial abundance and richness significantly increased in all six study lakes over the 500–1500 years reconstructed (depending on the sediment core). However, it was most evident after the arrival of Europeans in Lakes Hayes, Johnson, Pounui, and Rotoehu. Although the GLS models could not specifically test the relationship between land-use and cyanobacteria response, the exploration of the data suggests that both abundance and richness increased significantly post European settlement, particularly for Lakes Hayes and Johnson. Palynology data from the cores used in this study shows that there was a marked decrease in native forests in the catchments of Lakes Hayes, Johnson, and Rotoehu, coinciding with the periods of increasing cyanobacterial abundance.

All lakes presented in this study have some historical environmental/social records, though the degree of knowledge varies across lakes (summarised in Supplementary Table S12). In Lake Hayes, cyanobacteria abundance has increased steadily since European settlement (1862^85^) and especially since the early 1900s with the conversion of much of the lower catchment into sheep pasture. Furthermore, superphosphate fertiliser application began in the 1950s; by 1961 there had been significant drainage of the wetlands in the catchment, and by the 1970s Lake Hayes was considered eutrophic. This increase in trophic state aligned with the first reports of cyanobacteria blooms (1970–1972, *Dolichospermum flos-aquae*^48^), and our own observations of potentially toxic cyanobacteria in the second half of the Post-European Settlement phase (PES, top 20 cm of the core). Similarly, the catchment of Lake Johnson, near Lake Hayes, was spared from urban development, but forest clearing from c. 1895 led to pasture development and application of superphosphate fertiliser from c. 1955^48^. Subsequently, potentially toxic taxa appear continuously in the sediment core with European settlement (~ 45 cm), and in higher proportion in the top 8 cm. Cyanobacterial richness and abundance both increased with land-use intensification, which may be related to superphosphate application (1955). Lake Rotoehu, the northernmost lake of this study, receives phosphorus-rich waters from geothermal input(s), has numerous shallow arms and generally quite warm waters (min 8 °C, max 22 °C)^86^. Its name in Te Reo Māori translates as “murky/cloudy (ehu) lake (roto)”, which could suggest that phytoplankton biomass might already have been high when Māori were living around the lake. However, the data in the present study indicate that if this was the case, it was likely not due to cyanobacteria, as historic levels were relatively low. The first cyanobacterial blooms were observed in the 1960s, coinciding with the first changes in land-use (forest and scrubs converted to pasture)^86,87^. In the 1960–70s, Lake Rotoehu was considered mesotrophic but a marked decrease (4.2 m) in lake level in 1993 is thought to have increased nutrient levels and possibly enhanced cyanobacterial blooms, which have occurred on an annual basis since then^88^. While we do not have any dates for the core of Lake Rotoehu apart from the tephra (1886, 30.5 to 37 cm), potential bloom-forming species also appear continuously from 18.5 cm to the top.

The results from these three lakes are in agreement with previous studies that have indicated a link between land use change, increased nutrient runoff, and cyanobacterial blooms and associated toxins. For example, in Baptiste Lake (Alberta, Canada) an increase in microcystin gene copies was correlated with nutrient enrichment^42^. Similarly, increases in agriculture in the surrounding landscape of Anderson Lake (Washington, USA) were linked with the first appearance of toxic *Dolichospermum* blooms^89^, and saxitoxin gene copies increased in Laguna Blanca (Maldonado, Uruguay) after forestry developed in its catchment^90^. Further work is required to fully identify the drivers of change as the definition of land-use intensification was categorical (presence/absence) rather than a quantitative measure of change in the catchment and was highly colinear with non-native fish introduction. The analysis would be enhanced by including more lakes and more variables that were not available for all the lakes studied.

In contrast to the previous three lakes, Lake Pounui provides an interesting anomaly to the proposed link between land-use change and the development of cyanobacterial blooms. Palynological data^47^ and historic knowledge^91^ suggest that land clearance only occurred within a part of the catchment and mostly stopped after a few decades. Aerial photographs show that land clearing in the southern side of the catchment had already occurred in 1941^91^, but the native forest had mostly regenerated by 1961. A possible link to the severity of cyanobacteria blooms in this lake is the introduction of rainbow trout (*Oncorhynchus mykiss)* and the European perch (*Perca fluviatilis*) into Lake Pounui and associated streams and lakes in the wider catchment, from 1938 and the 1960s, respectively (Fish and Game NZ, historic records; Rawhiri Smith, pers. comm.).

Non-native fish can disrupt native food webs^92,93^ and have a top-down impact on cyanobacteria^94^. Pelagic food webs are quite simple in New Zealand, as they are lacking strongly piscivorous fish^95,96^. Voracious zooplanktivorous fish such as juvenile perch are absent from the indigenous fauna, and when introduced, they can effectively shift zooplankton communities so that smaller species (e.g. rotifers) become dominant, thus reducing grazing pressure on phytoplankton^97^. *Perca fluviatilis* have few effective predators in New Zealand, albeit they are cannibalistic, and, as such, their populations are often stunted and mostly composed of juveniles, therefore enhancing zooplanktivory and reducing grazing on phytoplankton^98^. Brown trout (*Salmo trutta*) and *perch* were introduced to Lake Hayes in the 1870s, while for Lake Johnson, *perch* was introduced in the 1880s and rainbow trout have been stocked annually since 1962. Lake Rotoehu also contains* rainbow trout*, which were introduced between 1898 and 1903^51^. Only a handful of studies have explored the trophic effects of perch *(P. fluviatilis)* introductions in New Zealand^99,100^, and more detailed food-web related research is required to fully understand the wider impacts of the introduction of these species on cyanobacterial blooms in these lakes.

Analysis of the metabarcoding data was required to further understand the recent increases in cyanobacterial abundance in Lakes Paringa and Wairarapa. The vegetation in the catchment of Lake Paringa is relatively unmodified^56^, therefore we anticipated there would be no change in cyanobacterial abundance at this site. Conversely, the catchment of Lake Wairarapa has been highly modified^50,57^ but only very sporadic and localised cyanobacterial blooms have been reported (e.g. *Dolichospermum lemmermannii* in 2008^50^). Lake Wairarapa is a very shallow lake with constant sediment resuspension; therefore any labile material could be thoroughly degraded before it settled down on the lakebed. This might have an impact on sedaDNA recovery and analysis. The metabarcoding data indicated that total increase in cyanobacteria copy numbers in these two lakes was due to picocyanobacteria. Picocyanobacteria are abundant in the oceans and often in oligotrophic lakes, but more recently they have also been reported in high numbers in eutrophic lakes, particularly coastal lakes and lagoons^101,102^. Picocyanobacteria are sensitive to high light intensities^103^, and the low-light conditions in both Lakes Paringa and Wairarapa (respectively brown waters due to tannins, and turbid waters from sediment resuspension) could potentially explain their abundance and dominance in these lakes. The increase in abundance in picocyanobacteria in Lake Paringa occurred in the 1960s. This coincides with the introduction of *S. trutta* (brown trout) in the 1950s, and the construction of a road in 1958 along the south-eastern side of the lake, thus enhancing human access to the lake. The dramatic increase in picocyanobacteria communities in the top 3 cm of the core in Wairarapa could suggest this is a recent change, possibly representing a further decline in the health of this lake.

### Other potential drivers of change

In addition to the impact of land-use change, and non-native fish introduction, many other factors impacted these lakes. For example, Lake Wairarapa have been significantly affected by hydrological changes, such as changing the main inflow away from the lake in the 1960s. Earthquakes can also have significant impacts on lakes, potentially triggering landslides, especially in mountainous terrain^104,105^. Landslides increase sediment yields directly in the lake, and tributaries flowing in to the lakes. The Alpine fault line near Lake Paringa has a magnitude 8 earthquake approximately every 300 years^56^, with at least four earthquakes occurring during the time period covered by the sediment core. Based on the results in this study, these earthquakes do not always appear to align with major shifts in cyanobacterial abundance or composition in Lake Paringa. One exception seems to be the 1717 Alpine fault earthquake, which is followed by a slight increase in cyanobacteria abundance. In 1855, a major earthquake^106^ (est. M 8.2) struck the Wairarapa region where Lake Wairarapa and Lake Pounui are situated, as well as smaller one in 1942^107^ (max M 7.2 and 6.8). In Lake Pounui, these events were concomitant with the discrete appearance of *Synechococcus* after these two events, and post 1855 there was a shift to *Dolichospermum* which is now dominating the cyanobacteria community. However, Europeans also started more intensive use of the land in the catchment around the same time and it is therefore not possible to correlate these changes in community structure with earthquakes specifically.

The impact of climate change was not assessed in this study because its timing overlaps with the main anthropogenic stressors studied here, making it extremely challenging to tease apart the impacts of climate change from other stressors. Further proxies which provide more detailed indications of climate change are required. Furthermore, focusing on lakes not affected by land-use, such as high-altitude alpine lakes, would yield more significant results. It is likely that climate change is enhancing the blooms. In New Zealand, average air temperatures have risen by 1.13 °C (± 0.27) from 1909 to 2019 nationally and there are more extreme, storm/rainfall events and longer periods of droughts since the beginning of the twentieth century^108^. It is extremely unlikely that this change in temperature alone is responsible for driving the changes in cyanobacterial abundance and composition observed in this study. If it was the case, blooms would be observed in Lake Paringa despite no major catchment changes, which is not the case.

### Caveats when interpreting sedimentary DNA data

Although sedaDNA approach are now increasingly used in paleolimnology, there are a number of caveats that need to be considered when interpreting the data. Many of these are well acknowledged challenges with the use of molecular approaches for analysing eDNA from a variety of sample matrices including primer biases, appropriate selection of universal (or targeted) primers and incomplete reference databases^33,109,110^.

One issue that requires further investigation in paleolimnological studies is DNA degradation. Degradation leads to DNA fragmentation; however it is currently unknown how quickly this occurs. Studies on contemporary samples indicate that amongst others, hight amounts of light (UV irradiance), high temperatures, acidic and oxic conditions contribute to DNA decay^111–116^. A recent study suggests that sedaDNA degradation mainly occurs over the first 250 years after sediment deposition^117^, however this research was undertaken in a tropical swamp (Uganda) and thus the environment conditions (especially temperature and pH) are very different to the lakes studied here. It is likely that degradation rates would be much slower rates in deeper, temperate lake sediments. DNA degradation in lake sediment cores needs further exploration, using methodologies such as shotgun sequencing which shows the degree of fragmentation in DNA sequences.

A puzzling observation in the present study was the higher ASV richness in all lakes in the PES phase, albeit this was most pronounced in the lakes that experienced the greatest increase in cyanobacterial abundance. This is somewhat contrary to expectations, given that cyanobacterial blooms are generally dominated by one or few species. There are several plausible explanations for this observation. Sediments core samples integrate time, they capture activity across an entire year and over multiple years of within lake biological activity. Cyanobacterial blooms primarily occur in summer months^118^, yet the sediment samples capture an annual signal, thus it is feasible that richness escalates during non-bloom periods. A study which focused on collecting regular water samples for at least a year is required to determine whether seasonal differences in cyanobacterial ASV abundance drive the observed increase in ASV richness. Additionally, in this study we took 1-cm wide depth slices for most lakes, which we estimate captures a period of 2–5 years in Lakes Hayes and Johnson, approximately 10 years in Lake Pounui, and 0.5 cm slices in Lake Paringa which captures about one year of time. During these most recent periods there have been rapid shifts in many environmental variables not measured in this study such as nutrients, chemicals, and toxins. It is feasible that the high species richness may truly represent a community that is adapting and diversifying to respond to an increasing number of new pressures. A further possibility is that DNA degradation is accounting for the reduction of richness in deeper samples. In metabarcoding studies a large proportion of ASVs are rare and have a low number of reads^119^. In this study 18 photosynthetic cyanobacterial ASVs (out of 839) accounted for 50% of the reads after rarefaction. As DNA deeper in the core is subjected to degradation pressures for a longer period, there is a likelihood that the rare ASVs are more likely to be lost compared to more recent samples, thus decreasing richness.

## Conclusion

Through the study of six different lakes over ~ 1000 years, this research has provided valuable new insights into changes in cyanobacterial communities in a range of lake types in New Zealand. Although cyanobacteria were present in these lakes prior to human arrival, including potentially toxic or bloom forming species, their overall abundance was low. Some changes in cyanobacterial abundance and structure occurred following Māori settlement in New Zealand, but the most pronounced shifts happened post European settlement, concomitant with land use change. The inclusion of Lakes Pounui and Paringa, where there has been only moderate (Pounui) or minimal (Paringa) land use change in the catchment, highlights that there are multiple drivers of shifts in cyanobacterial communities. While we speculate that the introduction of non-native carnivorous fish played a role in changing cyanobacterial communities, further work exploring multiple trophic levels is required. Our results demonstrate the high utility of combining ddPCR and metabarcoding for investigating shifts in cyanobacterial abundance and structure in lakes over many centuries. Paleolimnological studies utilising sedaDNA offer the potential to track the history of cyanobacteria, and when used in combination with other paleolimnological proxies the data can provide insights into the drivers of change.

## Supplementary Information

Supplementary Information.

## Data Availability

The metabarcoding dataset generated and analysed during the current study is available in the Sequence Read Archive (SRA) repository (BioProject PRJNA855252).
