# Supplementary material for: Using metabarcoding and droplet digital PCR to investigate drivers of historical shifts in cyanobacteria from six contrasting lakes
Source: Sci Rep. 2022 Jul 27;12:12810. doi: 10.1038/s41598-022-14216-8 (PMC9329365; doi:10.1038/s41598-022-14216-8)
Supplement: Supplementary file 1 — Supplementary Information. [file 41598_2022_14216_MOESM1_ESM.docx]

# Supplementary material

**Supplementary Table S1**: Sub-sampling depths per core and corresponding dates. Phases are indicated in shades of blue: Post-European Settlement (PES - light blue), Evidence of Māori Settlement (EMS - blue), Pre-Human (PH - dark blue).

| Paringa | | | Pounui | | Rotoehu | Hayes | Johnson | Wairarapa |
| --- | --- | --- | --- | --- | --- | --- | --- | --- |
| Depth (cm) | | Date | Depth (cm) | Date | Depth (cm) | Depth (cm) | Depth (cm) | Depth (cm) |
| 0 | | 2011 | 0-1 | 2013 | 0.5 | 0-1 | 0-1 | 0-1 |
| 2.5 | | 2007 | 1-2 | 1999 | 1.5 | 2-3 | 1-2 | 2-3 |
| 7.5 | | 1999 | 2-3 | 1992 | 2.5 | 4-5 | 2-3 | 4-5 |
| 12.5 | | 1989 | 3-4 | 1985 | 3.5 | 6-7 | 3-4 | 6-7 |
| 19.5 | | 1971 | 4-5 | 1978 | 4.5 | 8-9 | 4-5 | 8-9 |
| 29.5 | | 1964 | 5-6 | 1963 | 5.5 | 10-11 | 5-6 | 10-11 |
| 40 | | 1950 | 6-7 | 1956 | 6.5 | 12-13 | 6-7 | 12-13 |
| 67.5 | | 1873 | 7-8 | 1950 | 9.5 | 14-15 | 7-8 | 14-15 |
| 74.5 | | 1856 | 8-9 | 1937 | 12.5 | 16-17 | 8-9 | 16-17 |
| 82 | | 1837 | 9-10 | 1930 | 15.5 | 18-19 | 9-10 | 18-19 |
| 89 | | 1811 | 11-12 | 1911 | 18.5 | 20-21 | 10-11 | 20-21 |
| 99.5 | | 1782 | 13-14 | 1891 | 21.5 | 22-23 | 11-12 | 22-23 |
| 105 | | 1765 | 15-16 | 1878 | 24.5 | 24-25 | 12-13 | 30-31 |
| 110 | | 1754 | 17-18 | 1859 | 27.5 | 26-27 | 13-14 | 35-36 |
| 115 | | 1745 | 19-20 | 1845 | 37.5 | 28-29 | 14-15 | 40-41 |
| 124 | | 1726 | 21-22 | 1773 | 41.5 | 30-31 | 16-17 | 45-46 |
| 129 | | 1724 | 23-24 | 1746 | 45.5 | 32-33 | 18-19 | 50-51 |
| 153 | | 1716 | 25-26 | 1719 | 49.5 | 34-35 | 20-21 | 55-56 |
| 189 | | 1652 | 27-28 | 1693 | 53.5 | 36-37 | 22-23 | 60-61 |
| 197 | | 1599 | 29-30 | 1675 | 57.5 | 38-39 | 24-25 | 65-66 |
| 237.5 | | 1463 | 34-35 | 1613 | 62.5 | 40-42 | 26-27 | 70-71 |
| 299 | | 1392 | 39-40 | 1551 | 68.5 | 44-46 | 28-29 | 75-76 |
| 318.5 | | 1344 | 44-45 | 1489 | 74.5 | 48-50 | 30-31 | 80-81 |
| 339.5 | | 1303 | 49-50 | 1440 | 83.5 | 52-54 | 32-33 | 85-86 |
| 356.5 | | 1241 | 55-56 | 1383 | 89.5 | 56-58 | 34-35 | 90-91 |
| 430.5 | | 1151 | 60-61 | 1338 | 95.5 | 60-62 | 36-37 | 95-96 |
| 442.5 | | 1077 | 65-66 | 1273 | 101.5 | 66-68 | 38-39 | 105-106 |
| 452.5 | | 998 | 70-71 | 1200 | 107.5 | 70-72 | 40-42 | 115-116 |
| 482 | | 963 | 75-76 | 1127 | 114.5 | 74-76 | 44-46 | 125-126 |
| 515.5 | | 939 | 80-81 | 1074 | 121.5 | 78-80 | 48-50 | 135-136 |
| 534 | | 866 | 85-86 | 1028 | 127.5 | 82-84 | 52-54 | 145-146 |
| 552.2 | | 790 | 90-91 | 982 |  |  | 56-58 | 155-156 |
|  |  | | 95-96 | 929 |  |  | 60-62 | 165-166 |
|  |  | | 100-101 | 883 |  |  | 64-66 | 175-176 |
|  |  | | 105-106 | 834 |  |  | 68-70 | 185-186 |
|  |  | | 110-111 | 784 |  |  | 72-74 | 195-196 |
|  |  | | 115-116 | 727 |  |  | 76-78 |  |
|  |  | | 120-121 | 670 |  |  | 80-82 |  |
|  |  | | 125-126 | 612 |  |  |  |  |
|  |  | | 130-131 | 562 |  |  |  |  |

**Supplementary Table S2**: Description of non-molecular analyses performed on the cores to establish pre-human, evidence of Māori settlement, and post-European settlement phases. Core chronology was only done for Lakes Pounui and Paringa, while pollen and charcoal counts were undertaken on all six lakes. Note that the methods varied slightly depending on the lake, therefore for detailed methods see Howarth et al. (2012) for Lake Paringa, Cochrane (2017) for Lake Pounui, Khan et al. (in review) for Lakes Hayes and Johnson, Waters et al. (2018) for Lake Wairarapa.

| Type of analysis | Description and method |
| --- | --- |
| Core chronology (^14^C) | Carbon-14 activity was measured from terrestrial leaf macrofossils in Lakes Paringa and Pounui. Macrofossils were cleaned and pre-treated then converted to CO_2_ and measured by accelerator mass spectrometry. Age-depth modelling was conducted using a Bayesian framework with the OXCAL 4.4 program. Dates are presented in Table S4 and Table S5. |
| Pollen | Extraction was carried out from sediment samples using 10% hot hydrochloric acid and acetolysis (6-micron sieving). Exotic *Lycopodium* tablets were added to each sample for pollen concentration calculations. |
| Charcoal | Charcoal was counted as number of fragments, grouped by fragment size, and presented as concentration per cm^3^. |

References:

Howarth, Jamie D., et al. "Lake sediments record cycles of sediment flux driven by large earthquakes on the Alpine fault, New Zealand." *Geology* 40.12 (2012): 1091-1094.

Cochrane, Leise. "Reconstructing ecological change, catchment disturbance, and anthropogenic impact over the last 3000 years at Lake Pounui, Wairarapa, New Zealand." (2017).

Khan, Sami, et al. “Palaeolimnological evaluation of historical nutrient and food web contributions to the eutrophication of two monomictic lakes” (in review).

Waters, Sean, et al. 2018. Lake Wairarapa — understanding the ecological past and present for future management. Prepared for Greater Wellington Regional Council. Cawthron Report No. 3157. 61 p. plus appendices

**Supplementary Table S3**: Primers used in this study, with reagent mix and cycling condition details. ddPCR = droplet digital PCR, and HTS = High-Throughout Sequencing.

|  | Target | Amplicon length | Primer name | Sequence | Source | Reaction mix (one sample) | Cycling conditions |
| --- | --- | --- | --- | --- | --- | --- | --- |
| ddPCR | Total cyanobacteria - 16S rRNA | 282 bp | CYAN 108F  CYAN 377R | Forward:  5’-ACGGGTGAGTAACRCGTRA-3’ | Urbach et al, (1992) | - 0.2 µL of primer at 10 µM - 10 µL of 2×BioRad QX200™ ddPCR EvaGreen Supermix - 7.6 µL of DNA/RNA free water (Life Technologies) - 4 µL of diluted DNA | - 95°C for 5 min - 50 cycles   - 95°C for 30 s   - 56°C for 1 min - 4°C for 5 min - 90°C for 5 min |
|  |  |  |  | Reverse:  5’-CCATGGCGGAAAATTCCCC-3’ | Nübel et al. (1997) |  |  |
| HTS | Total cyanobacteria- 16S rRNA | 400 bp | CYB359-F  CYB784-R | Forward:  5’-GGGGAATYTTCCGCAATGGG-3’ | Nübel et al. (1997) | - 25 µL of AmpliTaq Gold® 360 Master Mix (Life Technologies) - 5 µL of 360 GC enhancer (Life Technologies) - 5 µl of BSA (Sigma) - 1 µL of primer at 10 µM - 10 µL of DNA/RNA free water (Life Technologies) - 1 µL of extracted sedDNA | - 95°C for 10 min - 35 cycles   - 95°C for 30 s   - 52°C for 30 s,   - 72°C for 1 min - 72°C for 7 min. |
|  |  |  |  | Reverse:  5’-ACTACWGGGGTATCTAATCCC-3’ |  |  |  |

***Supplementary Figure S1****: Selection of pollen and charcoal data from the top of the Lake Pounui core. Pinus, Salix, and Rumex are exotic plants indicators of European presence, charcoal is an indication of vegetation clearance in the catchment (Māori then European presence). Adapted from Cochrane (2017). This data was used to identify the three phases (separated by horizontal purple lines) used in this study: pre-human (PH; bottom), evidence of Māori settlement (EMS; middle), and post European settlement (PES; top).*
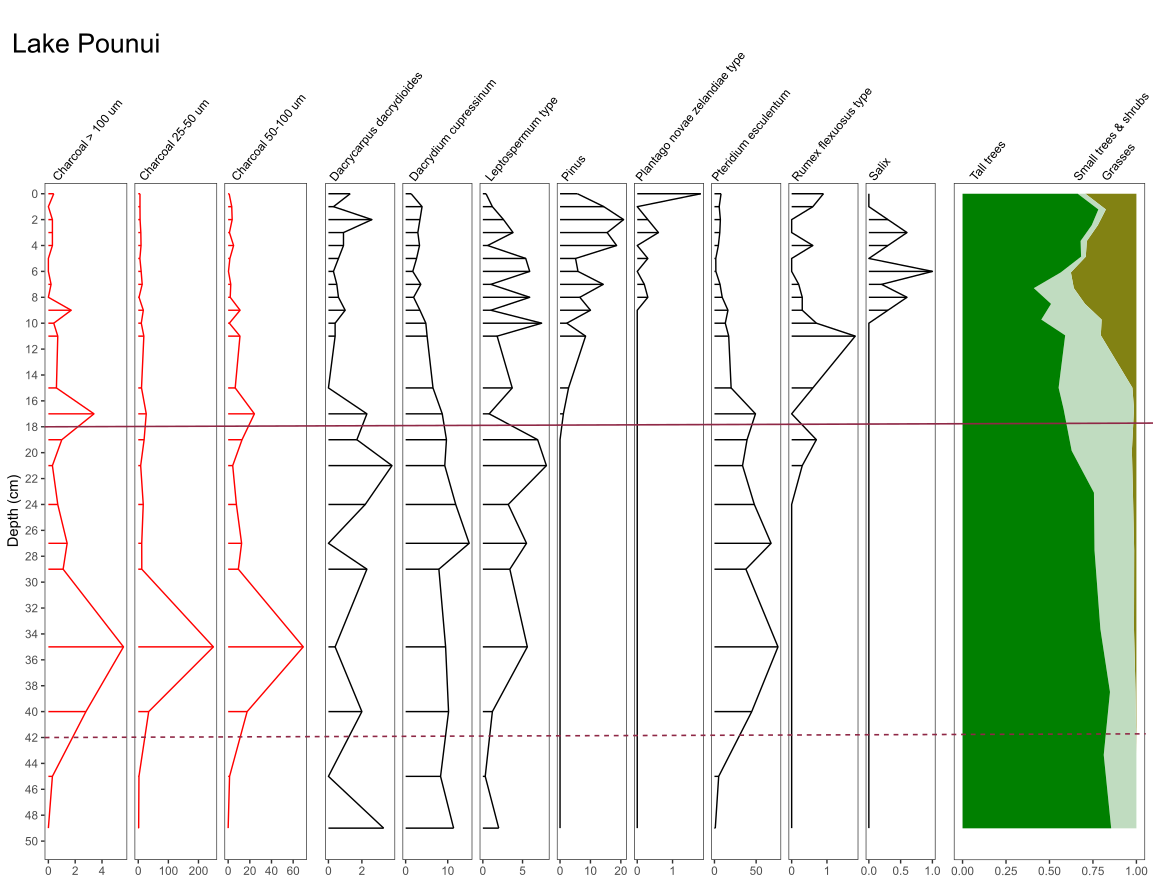


***Supplementary Figure S2****: Pollen and charcoal data from the Lake Rotoehu core. Pinus and Rumex are exotic plants indicators of European presence, charcoal is an indication of vegetation clearance in the catchment (Māori then European presence). Tephra layer indicates the 1886 eruption of Mount Tarawera. Data generated by the Lakes380 project (*[*www.lakes380.com*](http://www.lakes380.com)*). This data was used to identify the three phases (separated by horizontal purple lines) used in this study: pre-human (PH; bottom), evidence of Māori settlement (EMS; middle), and post European settlement (PES; top).*


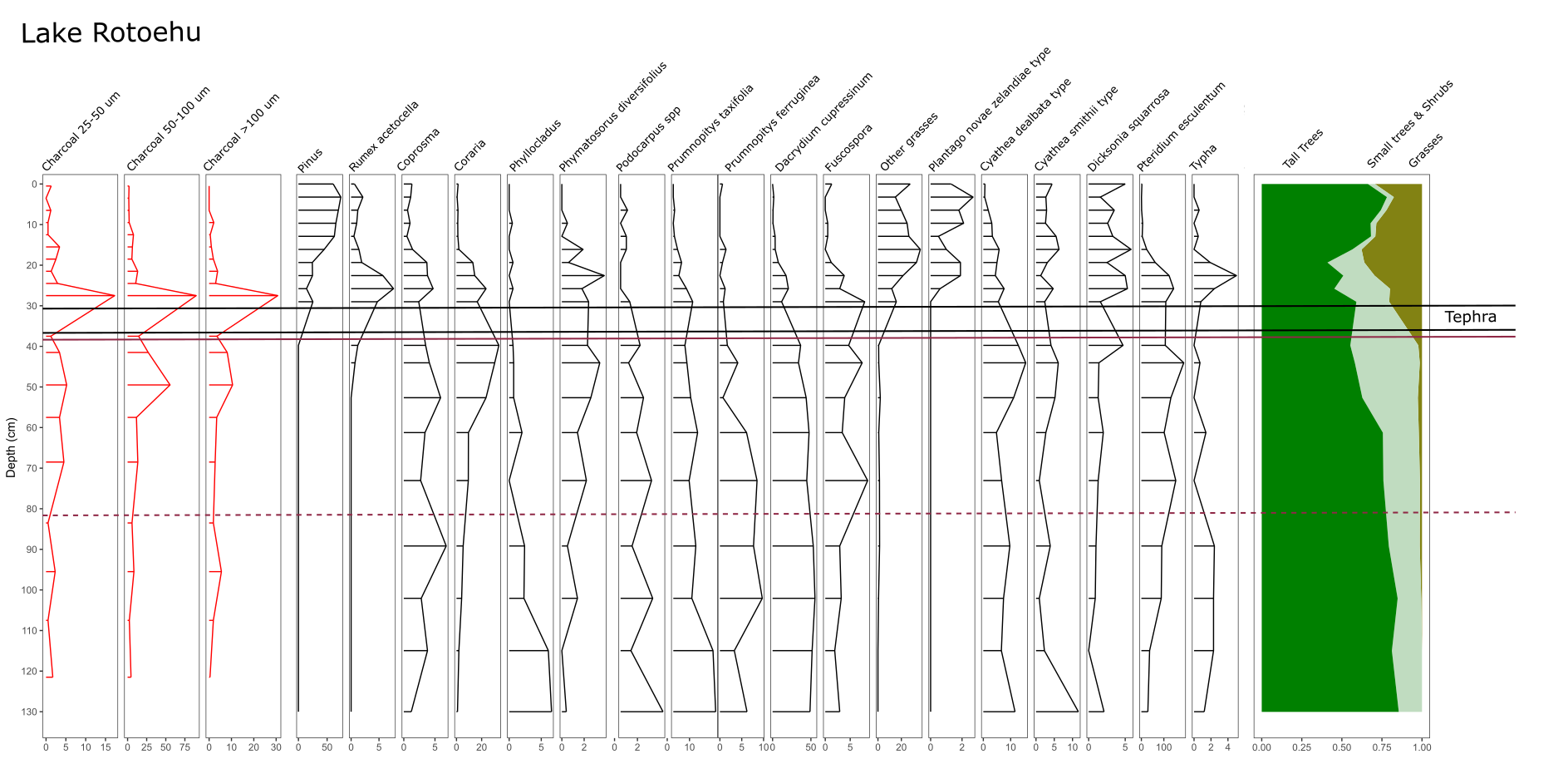


***Supplementary Figure S3****: Pollen and charcoal data from the Lake Hayes core. Pinus, Salix, and Rumex are exotic plants indicators of European presence, charcoal is an indication of vegetation clearance in the catchment. Adapted from Khan et al. (in review). This data was used to identify the three phases (separated by a horizontal purple line) used in this study: evidence of Māori settlement (EMS; bottom), and post European settlement (PES; top).*


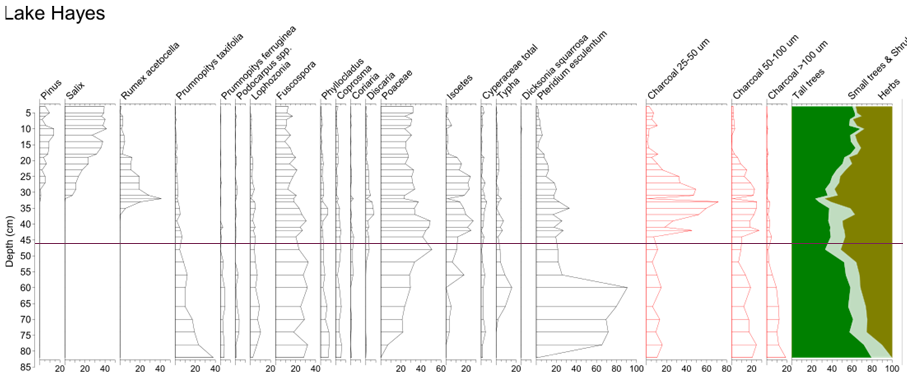


***Supplementary Figure S4****: Selection of pollen and charcoal data from the Lake Johnson core, used to determine occupation phases. Pinus, Salix, and Rumex are exotic plants indicators of European presence, charcoal is an indication of vegetation clearance in the catchment (Māori then European presence). Adapted from Khan et al. (in review). This data was used to identify the three phases (separated by horizontal purple lines) used in this study: pre-human (PH; bottom), evidence of Māori settlement (EMS; middle), and post European settlement (PES; top).*


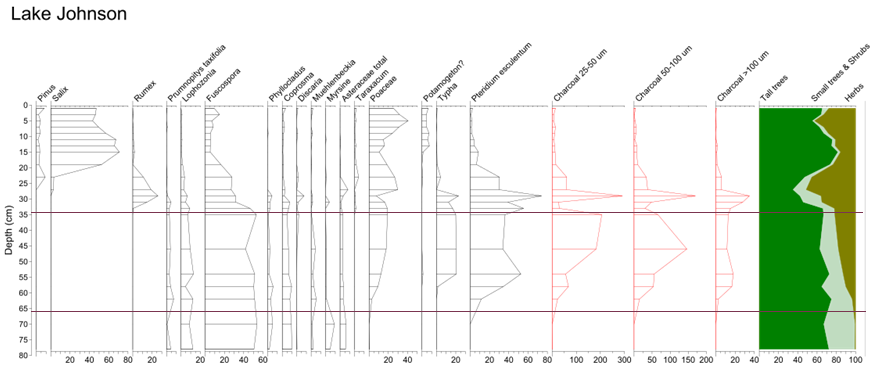


***Supplementary Figure S5****: Selection of pollen and charcoal data from the Lake Wairarapa core, used to determine occupation phases. Pinus, Salix, and Rumex are exotic plants indicators of European presence, charcoal is an indication of vegetation clearance in the catchment (Māori then European presence). Adapted from Waters et al. (2018). This data was used to identify the three phases (separated by horizontal purple lines) used in this study: pre-human (PH; bottom), evidence of Māori settlement (EMS; middle), and post European settlement (PES; top).*


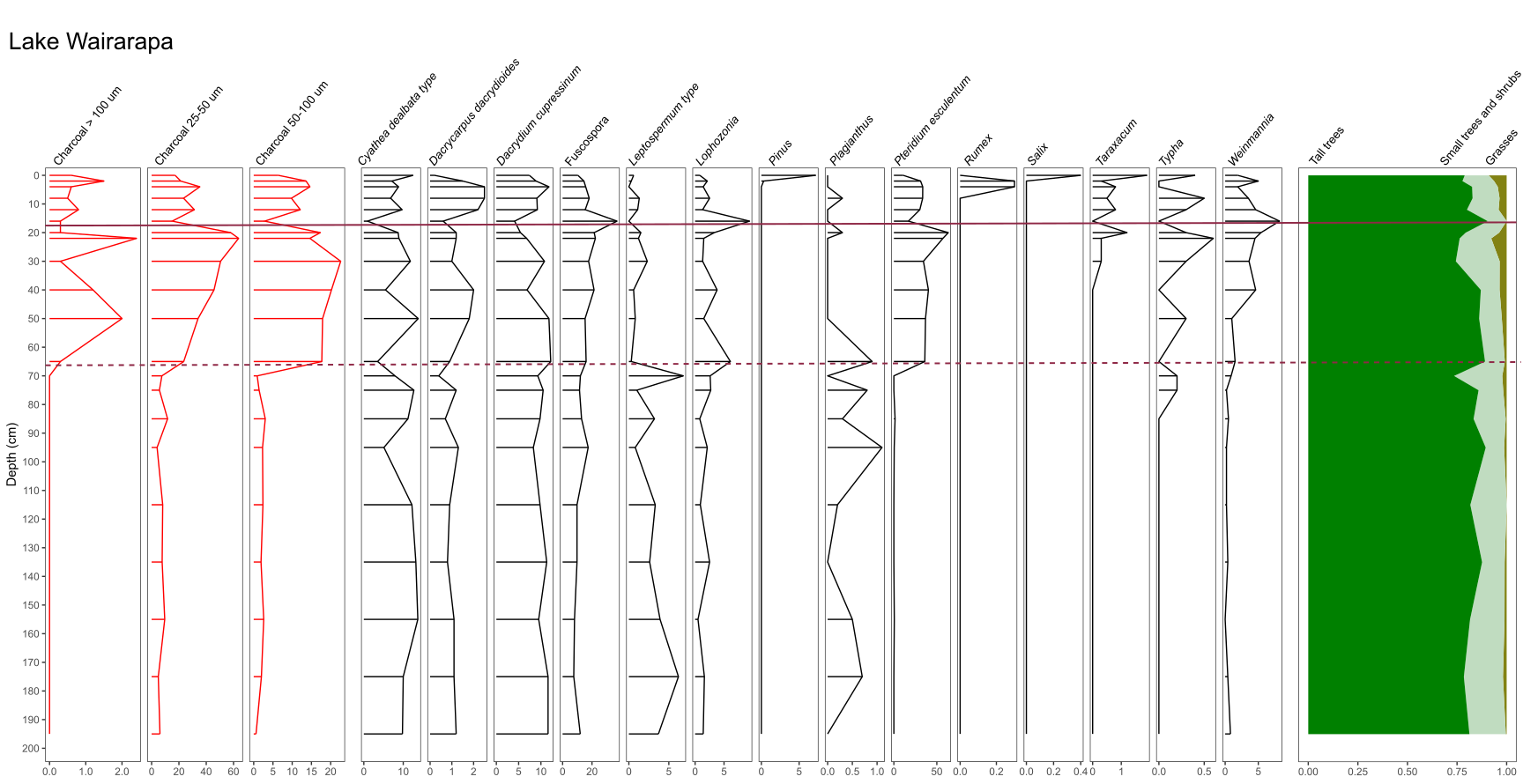


**Supplementary Table S4:** Chronology for Lake Paringa – adapted from Howarth et al. (2012).

| **Sample** | **Sub-bottom depth (cm)** | **RDL depth (cm)** | **Mean age (AD)** | **1 sigma uncertainty** | **Lower 2 sigma (AD)** | **Upper 2 sigma (AD)** |
| --- | --- | --- | --- | --- | --- | --- |
| PA1 3.5 cm D | 0 | 0 | 2011 | 0 | 2010 | 2011 |
| PA1 5.5 cm D | 1.5 | 1.5 | 2007 | 4 | 1995 | 2011 |
| PA1 10.5 cm D | 6.5 | 6.5 | 1999 | 8.3 | 1981 | 2011 |
| PA1 14.5 cm D | 11.5 | 11.5 | 1989 | 9.4 | 1969 | 2008 |
| PA1 25.0 cm D | 21 | 21 | 1971 | 7 | 1958 | 1986 |
| PA1 32.5 cm D | 29 | 27.5 | 1964 | 4.3 | 1952 | 1970 |
| PA1 43.5 D | 39 | 37 | 1950 | 6.3 | 1934 | 1960 |
| PA6 36 cm D | 68 | 63.5 | 1873 | 25.4 | 1821 | 1922 |
| PA6 43 cm D | 74 | 69.5 | 1856 | 26.3 | 1802 | 1908 |
| PA6 52 cm D | 80.5 | 76 | 1837 | 26.6 | 1783 | 1890 |
| PA6 59.5 cm D | 89.5 | 85 | 1811 | 25.7 | 1760 | 1863 |
| PA6 69.5 cm D | 99.5 | 95 | 1782 | 22.8 | 1738 | 1827 |
| PA6 76 cm D | 105.5 | 101 | 1765 | 19.8 | 1729 | 1804 |
| PA6 81.5 cm D | 110.5 | 105 | 1754 | 18.4 | 1720 | 1791 |
| PA6 87 cm D | 115 | 108 | 1745 | 17.2 | 1714 | 1780 |
| PA6 96.5 cm D | 125 | 115 | 1726 | 12.5 | 1702 | 1753 |
| PA6 101 cm D | 129.5 | 115.5 | 1724 | 12 | 1702 | 1751 |
| PA6 126 cm D | 153.5 | 118.5 | 1716 | 8.1 | 1700 | 1733 |
| PA6 S2 1.0 cm D | 189.5 | 132.5 | 1652 | 10.3 | 1632 | 1671 |
| PA6 S2 13.0 cm D | 201.5 | 144.5 | 1599 | 17.4 | 1558 | 1626 |
| PA6 S2 55 cm D | 238.5 | 167 | 1463 | 15.4 | 1436 | 1497 |
| PA6 S2 121.5 cm D | 300 | 187.5 | 1392 | 7.1 | 1374 | 1406 |
| PA6 S2 142.5 cm D | 319.5 | 200.5 | 1344 | 12.5 | 1319 | 1369 |
| PA6 S3 1.5 cm D | 336 | 213 | 1303 | 17.6 | 1266 | 1343 |
| PA6 S3 20.5 cm D | 357.5 | 233 | 1241 | 16.6 | 1212 | 1276 |
| PA6 S3 100 cm D | 431 | 253.5 | 1151 | 17.6 | 1109 | 1180 |
| PA6 S3 116 cm D | 442.5 | 264 | 1077 | 29.3 | 1024 | 1133 |
| PA6 S3 132cm D | 453.5 | 275 | 998 | 17.8 | 964 | 1030 |
| PA6 S4 15.5 cm D | 484 | 286 | 963 | 10.3 | 941 | 984 |
| PA6 S4 52 cm D | 517 | 291 | 939 | 27.7 | 865 | 974 |
| PA6 S4 74 cm D | 536.5 | 310.5 | 866 | 47.3 | 766 | 942 |
| PA6 S4 98 cm D | 558 | 330.5 | 790 | 63.1 | 659 | 901 |

**Supplementary Table S5:** Chronology for Lake Pounui. All ages are indicated in radiocarbon years (BP) except when specified (calendar years – AD). Adapted from Cochrane (2017).

| *Depth (cm)* | *Mean age* | *Mean AD age* | *sigma* | *Median age* | *Lower CI (95)* | *Upper CI (95)* | *Lower CI (99)* | *Upper CI (99)* |
| --- | --- | --- | --- | --- | --- | --- | --- | --- |
| 0 | -63 | 2013 | 1 | -63 | -61 | -66 | -61 | -66 |
| 1.625 | -49 | 1999 | 13 | -52 | -21 | -66 | -7 | -66 |
| 2.4375 | -42 | 1992 | 14 | -44 | -14 | -66 | -5 | -66 |
| 3.25 | -35 | 1985 | 15 | -35 | -7 | -61 | -2 | -66 |
| 4.0625 | -28 | 1978 | 15 | -26 | -4 | -59 | 0 | -66 |
| 5.6875 | -13 | 1963 | 10 | -10 | 1 | -39 | 1 | -61 |
| 6.5 | -6 | 1956 | 3 | -5 | 0 | -12 | 9 | -31 |
| 7.23125 | 0 | 1950 | 11 | -3 | 31 | -14 | 68 | -31 |
| 8.69375 | 13 | 1937 | 17 | 9 | 52 | -11 | 92 | -19 |
| 9.425 | 20 | 1930 | 20 | 16 | 62 | -9 | 97 | -15 |
| 11.6187 | 39 | 1911 | 23 | 38 | 84 | -4 | 106 | -9 |
| 13.8125 | 59 | 1891 | 23 | 60 | 103 | 12 | 116 | -6 |
| 15.275 | 72 | 1878 | 22 | 74 | 111 | 26 | 129 | -3 |
| 17.4688 | 91 | 1859 | 17 | 94 | 121 | 52 | 147 | 18 |
| 19 | 105 | 1845 | 11 | 105 | 126 | 81 | 161 | 71 |
| 21.2219 | 177 | 1773 | 27 | 173 | 241 | 106 | 303 | 89 |
| 23.3875 | 204 | 1746 | 36 | 199 | 293 | 147 | 359 | 101 |
| 25.5531 | 231 | 1719 | 42 | 226 | 323 | 157 | 393 | 112 |
| 27.7188 | 257 | 1693 | 46 | 253 | 353 | 171 | 424 | 133 |
| 29.1625 | 275 | 1675 | 48 | 272 | 375 | 183 | 439 | 150 |
| 34.2156 | 337 | 1613 | 49 | 338 | 435 | 237 | 470 | 189 |
| 39.2687 | 399 | 1551 | 43 | 406 | 475 | 307 | 491 | 245 |
| 44.575 | 461 | 1489 | 27 | 466 | 500 | 393 | 504 | 339 |
| 49.2 | 510 | 1440 | 8 | 510 | 528 | 495 | 544 | 481 |
| 55.6125 | 567 | 1383 | 25 | 568 | 616 | 518 | 635 | 502 |
| 60.6 | 612 | 1338 | 20 | 616 | 646 | 552 | 659 | 543 |
| 65.2125 | 677 | 1273 | 44 | 674 | 777 | 595 | 842 | 554 |
| 70.3375 | 750 | 1200 | 49 | 750 | 852 | 648 | 916 | 601 |
| 75.4625 | 823 | 1127 | 40 | 820 | 924 | 753 | 948 | 684 |
| 80.25 | 876 | 1074 | 40 | 868 | 961 | 811 | 1036 | 773 |
| 85.125 | 922 | 1028 | 45 | 918 | 1017 | 837 | 1084 | 809 |
| 90 | 968 | 982 | 49 | 965 | 1068 | 873 | 1125 | 836 |
| 95.6875 | 1021 | 929 | 49 | 1020 | 1126 | 926 | 1160 | 879 |
| 100.562 | 1067 | 883 | 47 | 1064 | 1161 | 981 | 1178 | 935 |
| 105.344 | 1116 | 834 | 50 | 1111 | 1207 | 1008 | 1266 | 985 |
| 110.031 | 1166 | 784 | 56 | 1166 | 1282 | 1056 | 1343 | 1003 |
| 115.305 | 1223 | 727 | 59 | 1224 | 1343 | 1103 | 1409 | 1037 |
| 120.578 | 1280 | 670 | 60 | 1282 | 1401 | 1156 | 1460 | 1084 |
| 125.852 | 1338 | 612 | 58 | 1341 | 1451 | 1217 | 1500 | 1131 |
| 130.539 | 1388 | 562 | 54 | 1393 | 1490 | 1276 | 1530 | 1178 |

**Supplementary Table S6**: Description of the variables considered in Generalised Linear Models against cyanobacteria abundances and richness.

| **Variable name** | **Description** | **Type** |
| --- | --- | --- |
| Depth | individual sample depths in cm (e.g., 0, 2, 4, 6…) | Continuous auto-correlated variable |
| Lake | individual lakes (Paringa, Pounui, Rotoehu, Hayes, Johnson, Wairarapa) | Categorical fixed variable |
| Phase | Time frame corresponding to the presence of absence of specific activities in/around the lake (PES, EMS, PH) | Categorical explanatory variable |
| Phase2 | pre-European settlement vs post-European settlement | Categorical (binary) explanatory variable |
| Exotic Fish | presence/absence of exotic fish introduced in each lake. Whenever possible it was aligned with known dates of introduction (H, J, Pa, Po), otherwise it was aligned with European settlement (R, W) | Categorical (binary) explanatory variable |
| Native Vegetation Change | before/after start of high charcoal peaks (land burning) | Categorical (binary) explanatory variable |
| Land-Use intensification | presence/absence of European-related land-use in lake catchment. Paringa was considered pristine | Categorical (binary) explanatory variable |

**Supplementary Table S7**: Categories attributed to each taxonomic entity at Genus level and plotted as Figure 6 (bubble plot).

| **Genus** | **Type** | **Toxic** |
| --- | --- | --- |
| *Aliterella* | > 3 µm | No |
| *Anabaena_XPORK15F* | > 3 µm | Potentially |
| *Annamia_HOs24* | > 3 µm | Potentially |
| *Aphanizomenon_NIES81* | > 3 µm | Potentially |
| *Calothrix_KVSF5* | > 3 µm | Potentially |
| *Calothrix_PCC-6303* | > 3 µm | Potentially |
| *CENA359* | > 3 µm | No |
| *Chroococcopsis* | > 3 µm | No |
| *Cuspidothrix_LMECYA_163* | > 3 µm | Potentially |
| *Cyanobium_PCC-6307* | Picocyanobacteria | No |
| *Cyanothece_PCC_7425* | > 3 µm | No |
| *Cylindrospermum_PCC-7417* | > 3 µm | Potentially |
| *Dolichospermum_NIES41* | > 3 µm | Potentially |
| *Geitlerinema_PCC-7105* | > 3 µm | Potentially |
| *Geminocystis_PCC-6308* | > 3 µm | No |
| *Gleocapsa* | > 3 µm | No |
| *HAVOmat113* | > 3 µm | No |
| *JSC-12* | > 3 µm | No |
| *Leptolyngbya_ANT.L52.2* | > 3 µm | Potentially |
| *Limnolyngbya_CHAB4449* | > 3 µm | No |
| *Microcoleus_SAG_1449-1a* | > 3 µm | Potentially |
| *Microcystis_PCC-7914* | > 3 µm | Potentially |
| *Nodosilinea_PCC-7104* | > 3 µm | No |
| *Nostoc_PCC-7524* | > 3 µm | Potentially |
| *Nostoc_PCC-8976* | > 3 µm | Potentially |
| *Oscillatoria_PCC-6304* | > 3 µm | Potentially |
| *Oscillatoria_SAG_1459-8* | > 3 µm | Potentially |
| *Phormidesmis_ANT.LACV5.1* | > 3 µm | No |
| *Phormidium_MBIC10003* | > 3 µm | Potentially |
| *Planktothrix_NIVA-CYA_15* | > 3 µm | Potentially |
| *Pseudanabaena_PCC-6802* | > 3 µm | No |
| *Pseudanabaena_PCC-7429* | > 3 µm | No |
| *Richelia_HH01* | > 3 µm | No |
| *Scytolyngbya_XSP1* | > 3 µm | No |
| *Snowella_0TU37S04* | > 3 µm | No |
| *Sphaerospermopsis_BCCUSP55* | > 3 µm | No |
| *SU2_symbiont_group* | > 3 µm | No |
| *Synechococcus_MBIC10613* | Picocyanobacteria | Potentially |
| *Synechocystis_BDHKU-20401* | Picocyanobacteria | Potentially |
| *Tychonema_CCAP_1459-11B* | > 3 µm | Potentially |
| Unknown Cyanobacteriaceae | > 3 µm | No |
| Unknown Cyanobacteriales | > 3 µm | No |
| Unknown Cyanobiaceae | Picocyanobacteria | No |
| Unknown Eurycoccales | > 3 µm | No |
| Unknown Leptolyngbyaceae | > 3 µm | No |
| Unknown Microcystaceae | > 3 µm | Potentially |
| Unknown Nostocaceae | > 3 µm | Potentially |
| Unknown Oscillatoriaceae | > 3 µm | No |
| Unknown Synechococcaceae | Picocyanobacteria | No |
| Unknown Synechococcales | Picocyanobacteria | No |
| Unknown Xenococcaceae | > 3 µm | No |
| *Xenococcus_CRM* | > 3 µm | No |


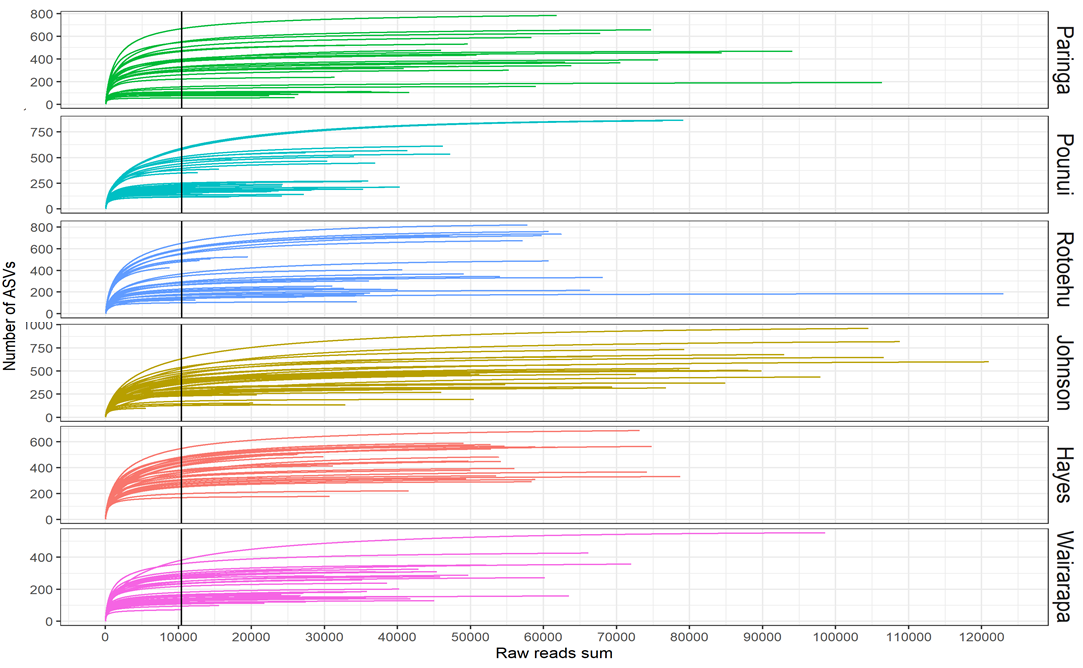


**Supplementary Figure S6**: Individual rarefaction curves, calculated on all bacterial reads. The vertical lines show the rarefaction threshold at 10,400 reads for univariate data (richness analyses).


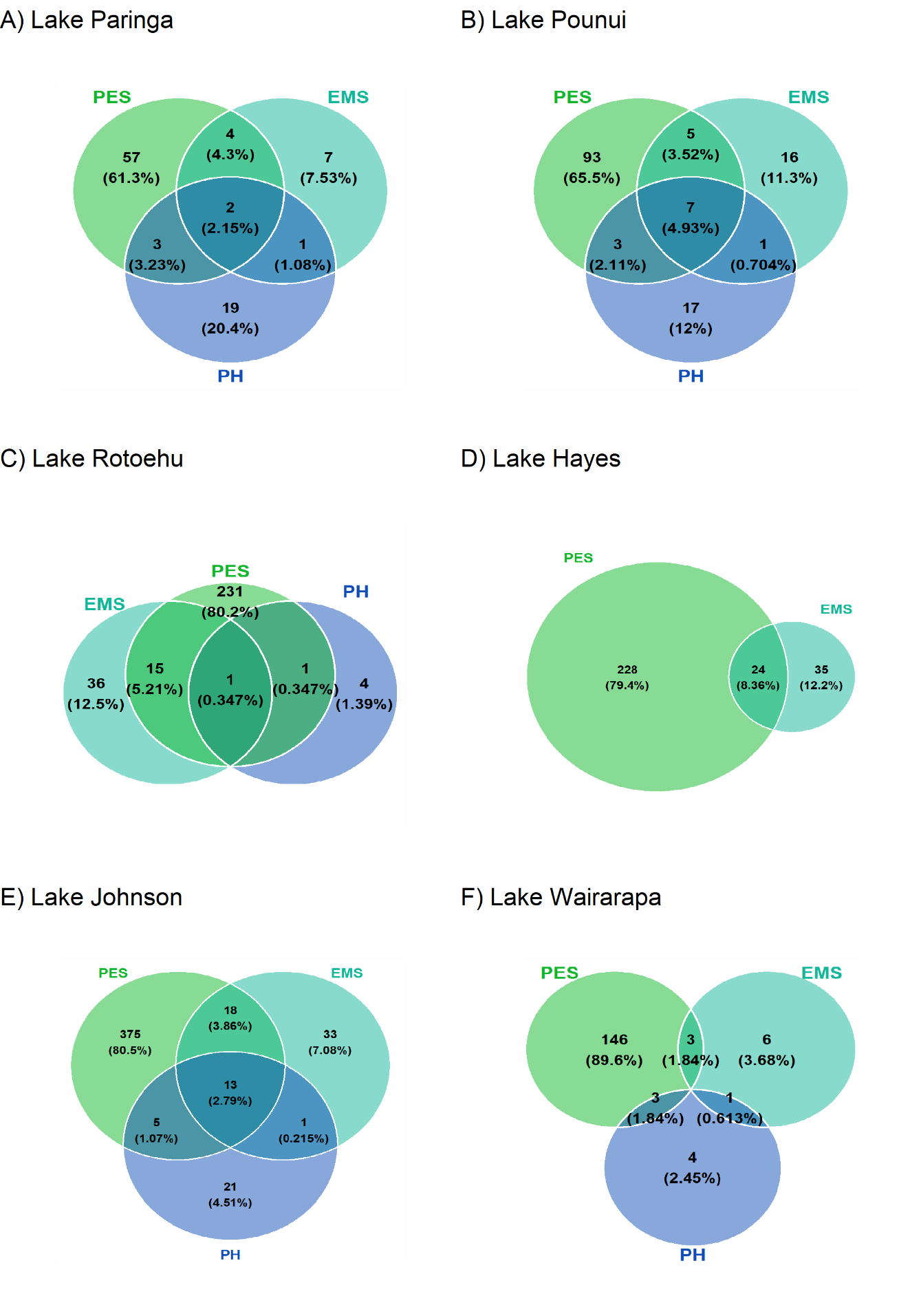


**Supplementary Figure S7**: Venn diagrams per lake showing the number and percentages of shared and unique Amplicon Sequence Variants across phases. Phases anagrams refer to occupation phases: PH = Pre-Human, EMS = Evidence of Māori Settlement, PES = Post-European Settlement. Drawn with the VennDiagram R package (Chen 2018).


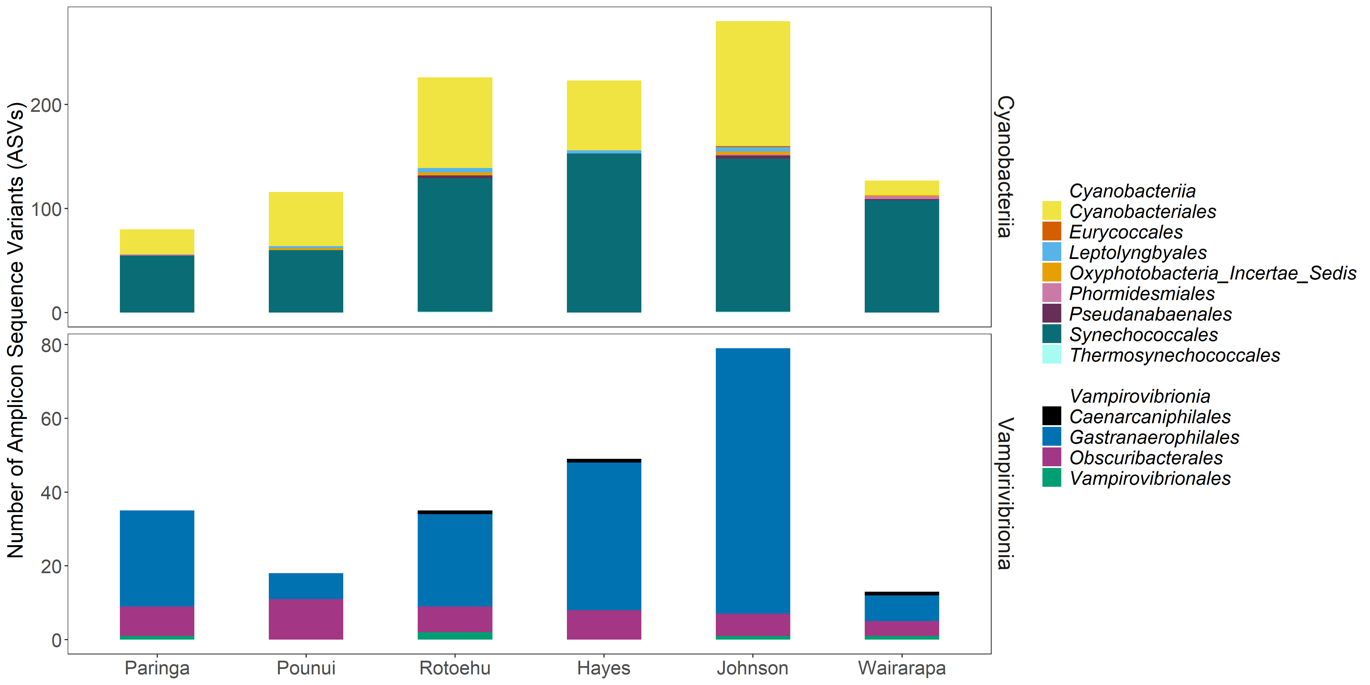


**Supplementary Figure S8**: Taxonomic composition and richness (number of Amplicon Sequence Variants) per lake at Class level and corresponding detail of Order level per Class. Since Cyanobacteriia is twice as abundant as Vampirovibrionia, different scales are used to improve visualisation.


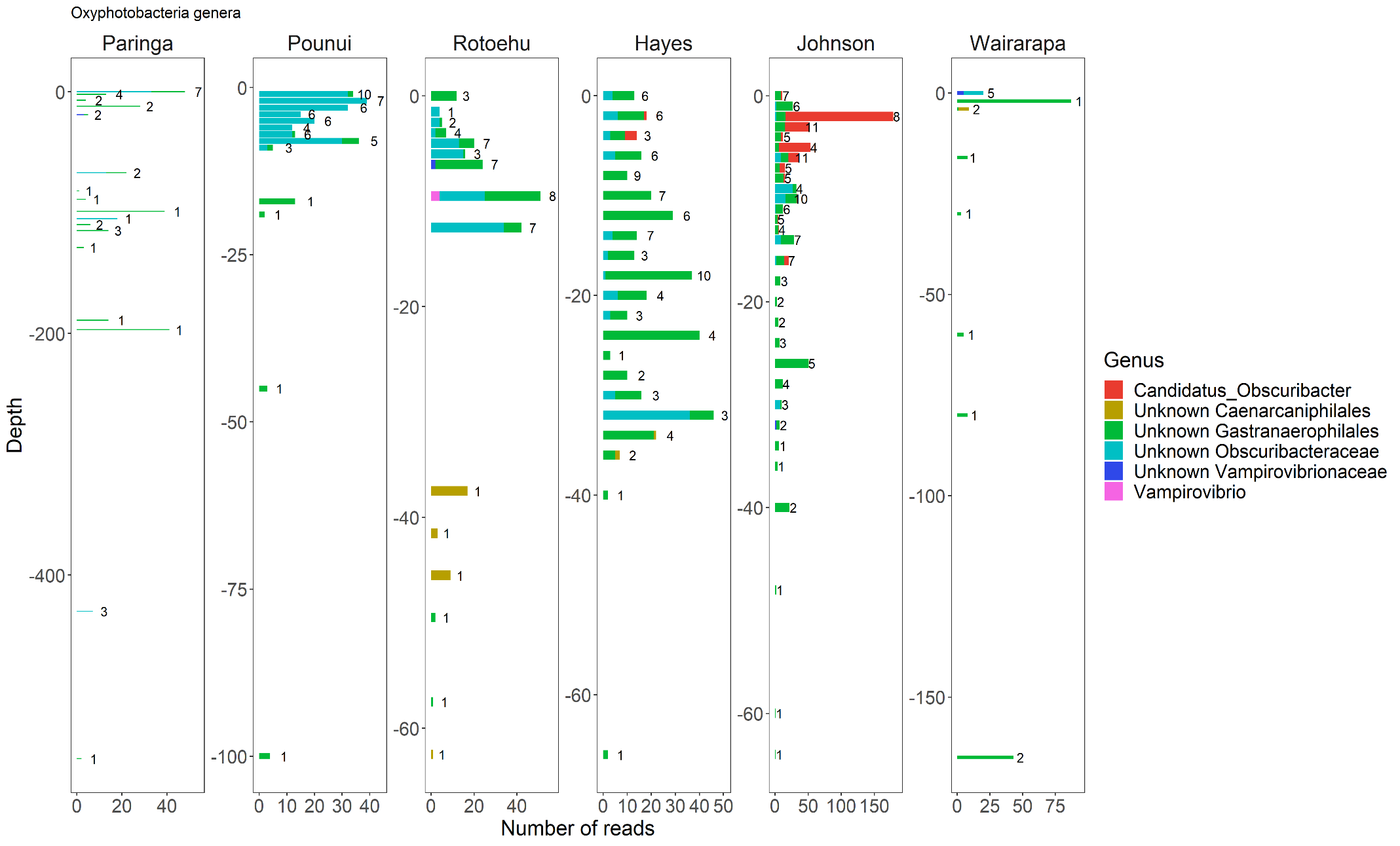


**Supplementary Figure S9**: Abundance of non-photosynthetic cyanobacteria (Class Vampirovibrionia) for each sample. Composition at Genus level (or closest resolved taxonomical level) is color-coded, and the number of Amplicon Sequence Variants (ASV) per sample (richness) is indicated on the right of each bar.


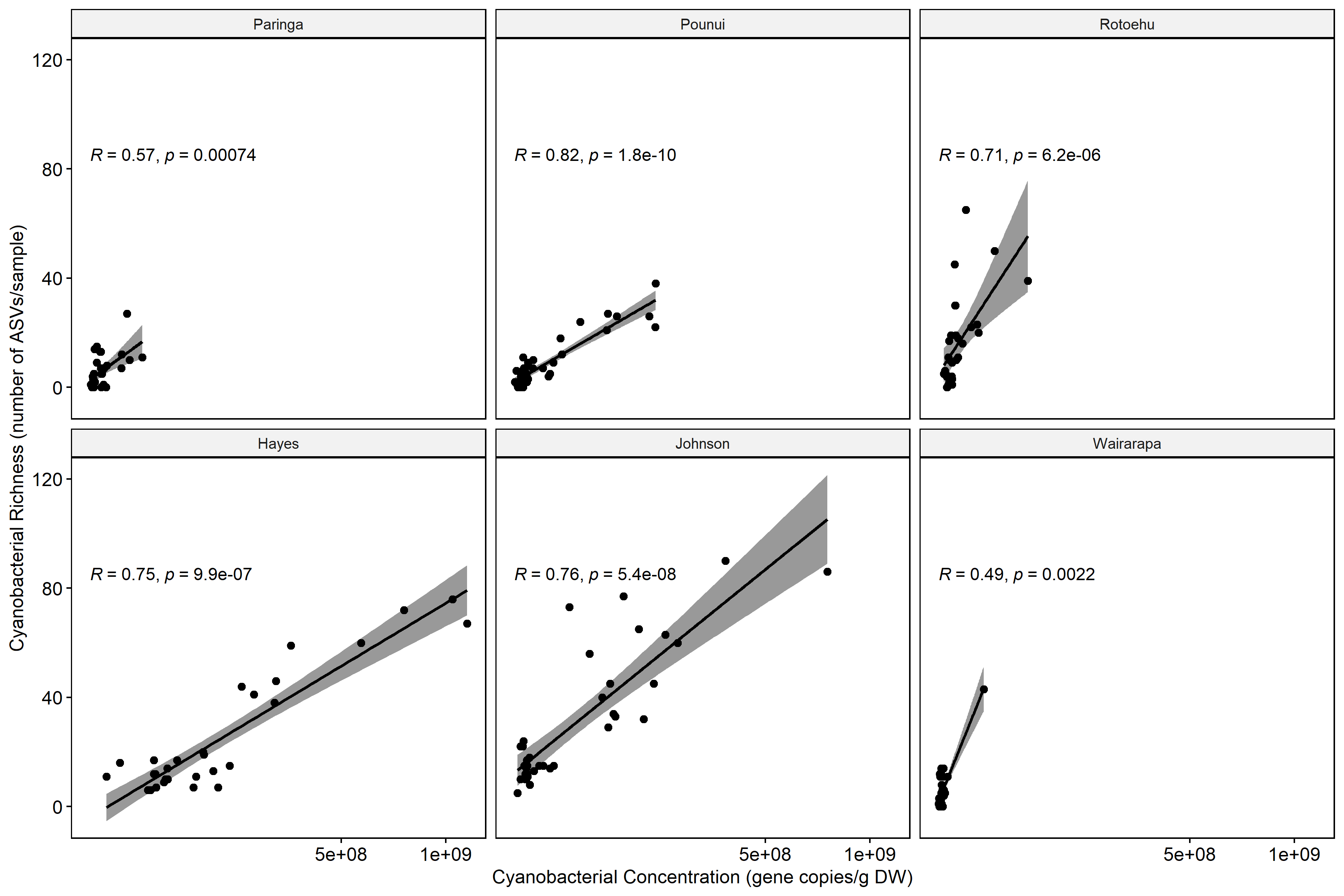


**Supplementary Figure S10**: Relationship between metabarcoding richness (number of amplicon sequence variants (ASVs)) and cyanobacterial abundance (16S rRNA cyanobacterial gene copy numbers per gram of dry sediment), lakes plotted individually, x-axis was transformed with square-root.

**Supplementary Table S8**: Lake pairwise comparisons of cyanobacterial abundance (16S rRNA gene, droplet digital PCR). Results were averaged by Phase and given on the log scale, degrees-of-freedom method was Satterthwaite and p-value adjustment method was tukey.

| **contrast** | **estimate** | **std.error** | **df** | **statistic** | **adj.p.value** |
| --- | --- | --- | --- | --- | --- |
| Paringa - Hayes | -4.28442 | 0.487311 | 56.51464 | -8.79196 | **< 0.001** |
| Paringa - Johnson | -1.62373 | 0.48031 | 70.05239 | -3.38058 | **0.01** |
| Paringa - Pounui | -1.43915 | 0.428028 | 42.00901 | -3.36228 | **0.02** |
| Paringa - Rotoehu | -1.12957 | 0.427571 | 42.81542 | -2.64184 | 0.11 |
| Paringa - Wairarapa | 1.076898 | 0.396292 | 55.62571 | 2.717437 | 0.09 |
| Hayes - Johnson | 2.660692 | 0.568402 | 85.74592 | 4.681005 | **< 0.001** |
| Hayes - Pounui | 2.845272 | 0.551843 | 63.00539 | 5.155947 | **< 0.001** |
| Hayes - Rotoehu | 3.154846 | 0.533699 | 62.75399 | 5.911286 | **< 0.001** |
| Hayes - Wairarapa | 5.361317 | 0.527012 | 87.28901 | 10.17305 | **< 0.001** |
| Johnson - Pounui | 0.18458 | 0.535947 | 81.58546 | 0.344399 | 0.99 |
| Johnson - Rotoehu | 0.494154 | 0.528002 | 84.91633 | 0.935894 | 0.94 |
| Johnson - Wairarapa | 2.700625 | 0.511749 | 76.64022 | 5.277243 | **< 0.001** |
| Pounui - Rotoehu | 0.309574 | 0.483094 | 67.36705 | 0.640816 | 0.99 |
| Pounui - Wairarapa | 2.516045 | 0.440256 | 48.17593 | 5.714955 | **< 0.001** |
| Rotoehu - Wairarapa | 2.206471 | 0.456369 | 50.91589 | 4.834834 | **< 0.001** |

**Supplementary Table S9**: Lake pairwise comparisons of cyanobacterial richness (16S rRNA gene, metabarcoding). Results were averaged by Phase and given on the sqrt scale, degrees-of-freedom method was Satterthwaite and p-value adjustment method was tukey.

| **contrast** | **estimate** | **std.error** | **df** | **statistic** | **adj.p.value** |
| --- | --- | --- | --- | --- | --- |
| Paringa - Hayes | -1.76359 | 0.65456 | 26.54497 | -2.69432 | 0.11 |
| Paringa - Johnson | -2.56889 | 0.658925 | 56.12537 | -3.89861 | **<0.01** |
| Paringa - Pounui | -0.61988 | 0.564298 | 9.29285 | -1.0985 | 0.87 |
| Paringa - Rotoehu | -0.90071 | 0.560841 | 9.949678 | -1.606 | 0.61 |
| Paringa - Wairarapa | -0.2071 | 0.50584 | 8.759694 | -0.40942 | 0.99 |
| Hayes - Johnson | -0.8053 | 0.806557 | 33.68649 | -0.99845 | 0.92 |
| Hayes - Pounui | 1.143706 | 0.763921 | 25.31611 | 1.497152 | 0.67 |
| Hayes - Rotoehu | 0.862882 | 0.740507 | 23.94373 | 1.165258 | 0.85 |
| Hayes - Wairarapa | 1.556489 | 0.721803 | 28.33236 | 2.15639 | 0.29 |
| Johnson - Pounui | 1.949011 | 0.758587 | 22.10189 | 2.569266 | 0.15 |
| Johnson - Rotoehu | 1.668187 | 0.745508 | 20.6873 | 2.23765 | 0.26 |
| Johnson - Wairarapa | 2.361794 | 0.717055 | 24.39192 | 3.293743 | **0.03** |
| Pounui - Rotoehu | -0.28082 | 0.66811 | 13.05836 | -0.42033 | 0.99 |
| Pounui - Wairarapa | 0.412782 | 0.606933 | 12.66998 | 0.680111 | 0.98 |
| Rotoehu - Wairarapa | 0.693607 | 0.620543 | 13.40042 | 1.117742 | 0.87 |


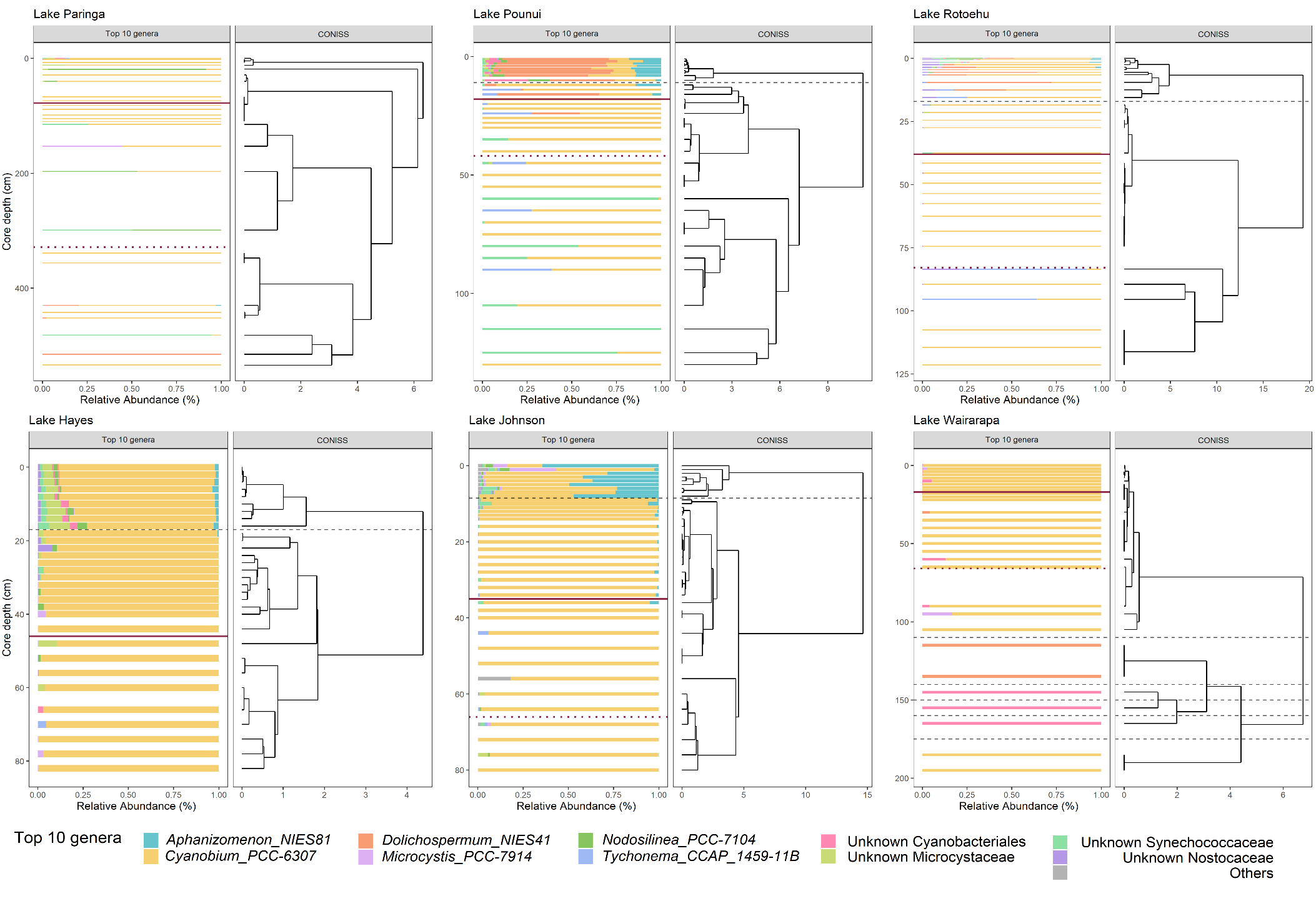


**Supplementary Figure S11**: Cyanobacteria composition at Genus level and CONISS analysis per lake. Only the top 10 genera are shown, the rest were grouped as “Others”. Occupation phases defined by the pollen data are indicated for each lake on the barplots (purple full line for European settlement, purple dotted line for Māori settlement). The CONISS analysis also identified main eras for all lakes except Lake Paringa, these are indicated by the grey dotted line going across the barplot and the dendrogram.

| 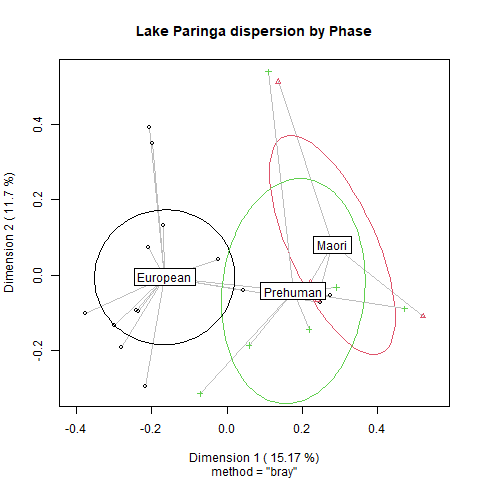 | 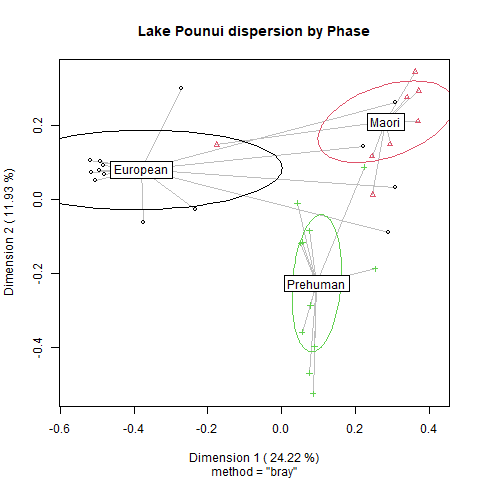 |
| --- | --- |
| 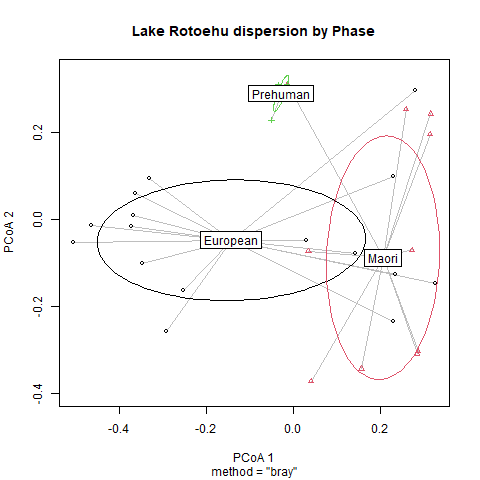 | 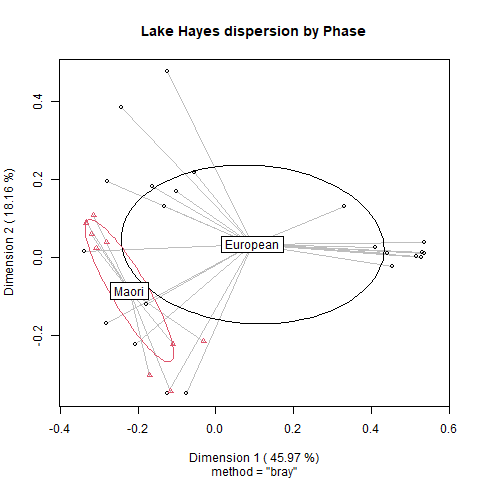 |
| 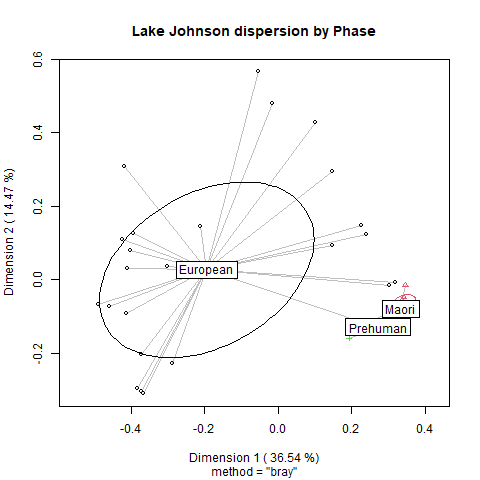 | 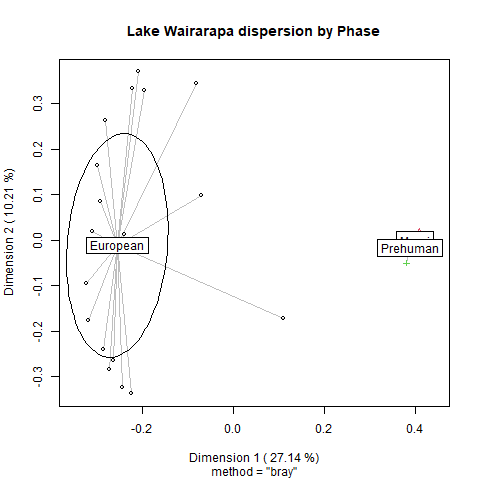 |

**Supplementary Figure S12**: Dispersion plots from the betadisper analysis of the R vegan package for phases within individual lakes.

**Supplementary Table S10**: PERMANOVA and pairwise test on cyanobacterial communities across phases within individual lakes. Since the core from Lake Hayes only retraced PES and EMS, no pairwise comparisons were needed (grey shading).

|  | PERMANOVA | | | Pairwise test | | |
| --- | --- | --- | --- | --- | --- | --- |
|  | F value | R^2^ | p-value |  | PES | EMS |
| Paringa | 1.9099 | 0.14 | **< 0.001** | EMS | **0.003** | - |
|  |  |  |  | PH | **0.002** | 0.81 |
| Pounui | 4.4398 | 0.22 | **< 0.001** | EMS | **0.004** | **-** |
|  |  |  |  | PH | **< 0.001** | **< 0.001** |
| Rotoehu | 2.8506 | 0.17 | **< 0.001** | EMS | **0.007** | **-** |
|  |  |  |  | PH | **< 0.001** | **0.006** |
| Hayes | 4.2593 | 0.13 | **0.005** |  |  |  |
|  |  |  |  |  |  |  |
| Johnson | 5.3147 | 0.23 | **< 0.001** | EMS | **< 0.001** | **-** |
|  |  |  |  | PH | **0.003** | **0.027** |
| Wairarapa | 5.9314 | 0.31 | **< 0.001** | EMS | **< 0.001** | - |
|  |  |  |  | PH | **< 0.001** | 0.18 |

**Supplementary Table S11**: ANOVA and Tukey multiple comparisons of means, testing the homogeneity of multivariate dispersions in cyanobacterial communities across phases within individual lakes. Since the core from Lake Hayes only retraced PES and EMS, no pairwise comparisons were needed (grey shading).

|  | ANOVA | | Tukey | | |
| --- | --- | --- | --- | --- | --- |
|  | F value | p-value |  | PES | EMS |
| Paringa | 6.7255 | **< 0.01** | EMS | **0.04** | - |
|  |  |  | PH | **0.02** | 0.99 |
| Pounui | 4.3828 | **0.02** | EMS | 0.48 | - |
|  |  |  | PH | **0.02** | 0.19 |
| Rotoehu | 5.4046 | **0.01** | EMS | 0.77 | - |
|  |  |  | PH | **< 0.01** | 0.06 |
| Hayes | 9.421 | **< 0.01** |  |  |  |
|  |  |  |  |  |  |
| Johnson | 22.421 | **< 0.001** | EMS | **< 0.001** | - |
|  |  |  | PH | **< 0.001** | 0.99 |
| Wairarapa | 23.738 | **< 0.001** | EMS | **< 0.001** | - |
|  |  |  | PH | **< 0.001** | 0.99 |

**Supplementary Table S12**: Summary of known events / lake states from the literature. European settlement started in 1840 after the signature of the Treaty of Waitangi.

|  | **Lake Paringa** | **Lake Pounui** | **Lake Rotoehu** |
| --- | --- | --- | --- |
| Post-European Settlement | Present day: lake open to the public, used for trout fishing. Pristine native catchment, dark waters (tannin). Oligotrophic.  1962-1965: Main road built next to lake.  1930: Brown trout (*Salmo trutta)* introduction.  1875: Haast Paringa cattle track (rough track used to move stock) provides some access to lake. | Present day: land around lake privately owned, cyanobacterial blooms mostly summer. Tens of thousands of perch (*Perca fluviatilis*) estimated to live in the lake. One of the last lowland lakes with a mostly native catchment and high native macrophyte presence/diversity. Eutrophic.  1970s: flood gates downstream found to prevent fish passage back to Lake Pounui.  1960s: perch (*Perca fluviatilis*) introduction.  1941: south-western catchment cut down (aerial image).  1938 to 1938: yearly stocking of fingerlings rainbow trout.  1855 – M 8.2: earthquake on nearby fault. | Present day: lake open to the public, recreational use. Cyanobacterial blooms every summer since 1993, especially in the multiple shallow arms. Eutrophic.  1993: marked decrease in lake level (4.2 m).  1960s: mesotrophic state, first land-use changes (forest and scrubland converted to pasture), first cyanobacterial blooms observed.  1900s: introduction of rainbow trout (*Oncorhynchus mykiss*).  1886 – eruption of Mount Tarawera covered the region with ash (c. 5cm thick tephra in core). |
| Evidence of Māori Settlement | No known Māori settlement – no evidence of land clearance.  Lake is situated near the Paringa fault: repeated earthquakes every c. 300 years.  Last earthquakes > 7.6 magnitude: c. AD 925, c. AD 1150, c. AD 1400 and AD 1717. | Māori settlement in the region estimated to c. 1500s-1600s (some land clearance) but no major settlement in the vicinity of the lake. | Māori settlement in the Rotorua region estimated to c. 1250s but no major settlement in the vicinity of the lake. |
| Pre-human |  | Lake existed in high nutrient state (before 0 AD) and then lower nutrient state (after 0 AD) until European arrival (diatom reconstruction, Cochrane 2017 MSc thesis). | Lake situated near active volcanoes. Long history of so impact from eruptions expected (tephra layers observed). |

|  | **Lake Johnson** | **Lake Hayes** | **Lake Wairarapa** |
| --- | --- | --- | --- |
| Post-European Settlement | Present day: lake open to the public, popular for trout fishing. Surrounded by pasture. Eutrophic. Contains rainbow trout.  1962: known beginning of cyanobacterial blooms. Also start of yearly stocking of fingerlings rainbow trout (*Oncorhynchus mykiss*).  1955: beginning of superphosphate fertiliser application in the catchment.  (unknown date) original outlet dammed to ensure water supply for irrigation.  1870s: Brown trout (*Salmo trutta*) and perch (*Perca fluviatilis*) introduced. | Present day: lake open to the public, popular for recreational (swimming, boating, fishing). Surrounded by pasture and a few settlements. Eutrophic.  2006: Blooms of *Ceratium hirundinella.*  1970: Bottom waters became anoxic, releasing phosphate. Now eutrophic.  1969: First cyanobacteria bloom recorded (*Dolichospermum flos-aquae*).  1961: Significant catchment drainage work began.  1959: Lake approaching eutrophic state.  1910-50: Increasing land-use around the lake (cheese factory releasing effluents, improved farming practices spreading superphosphate fertiliser).  1870s – Brown trout (*Salmo trutta*) and perch (*Perca fluviatilis*) introduced. | Present day: lake open to the public, very degraded state (super-eutrophic). Surrounded by pastures and a few towns. Supertrophic.  1963-1983 – Construction to prevent regular flooding of the valley (Lower Wairarapa Valley Development Scheme). Inflow (Ruamahanga River) diverted permanently from Lake Wairarapa.  1855 – M 8.2 earthquake on nearby fault.  Increase in burning, deforestation, agricultural land-use.  1844 - European settlement. |
| Evidence of Māori Settlement | No major Māori settlement. | No major Māori settlement. | Māori settlement in the region estimated to c. 1500s. |
| Pre-human |  |  |  |
